# DiSTect: a Bayesian model for disease-associated gene discovery and prediction in spatial transcriptomics

**DOI:** 10.1093/bioinformatics/btaf530

**Published:** 2025-09-22

**Authors:** Qicheng Zhao, Anji Deng, Qihuang Zhang

**Affiliations:** Department of Epidemiology, Biostatistics and Occupational Health, McGill University, Montreal, Quebec H3A 1G1, Canada; Department of Epidemiology, Biostatistics and Occupational Health, McGill University, Montreal, Quebec H3A 1G1, Canada; Department of Epidemiology, Biostatistics and Occupational Health, McGill University, Montreal, Quebec H3A 1G1, Canada

## Abstract

**Motivation:**

Identifying disease-indicative genes is critical for deciphering disease mechanisms and has attracted significant interest in biomedical research. Spatial transcriptomics offers unprecedented insights for the detection of disease-associated genes by enabling within-tissue contrasts. However, this new technology poses challenges for conventional statistical models developed for RNA-sequencing, as these models often neglect the spatial corrleation of the disease status among tissue spots.

**Results:**

In this article, we propose DiSTect, a Bayesian shrinkage model to characterize the relationship between high-dimensional gene expressions and the disease status of each tissue spot, incorporating spatial correlation among these spots through autoregressive terms. Our model adopts a hierarchical structure to facilitate the analysis of multiple correlated samples and is further extended to accommodate the missing data within tissues. To ensure the model’s applicability to datasets of varying sizes, we carry out two computational frameworks for Bayesian parameter estimation, tailored to both small and large sample scenarios. Simulation studies are conducted to evaluate the performance of the proposed model. The proposed model is applied to analyze the data arising from studies of HER2+ breast cancer and Alzheimer’s disease.

**Availability and implementation:**

The dataset and source code are available on GitHub (https://github.com/StaGill/DiSTect) and Zenodo (https://zenodo.org/records/17127211).

## 1 Introduction

Complex diseases often involve dysregulation at the cellular level that may not be uniformly distributed across tissues. To decipher the functions of cells in multicellular organisms and the mechanisms of complex diseases, understanding the relationship between high-dimensional gene expression data and disease status is crucial. The initial sequencing approaches were conducted on bulk tissue (Li and Wang 2021). Although economical, these approaches lacked the resolution to capture the heterogeneity of disease status within tissues. In contrast, single-cell RNA-seq (scRNA-seq) profiles gene expression in a single-cell resolution, providing a within-tissue contrast of disease status (Sant *et al.* 2023). Such a contrast naturally introduces a matched design, bringing the opportunity to control for potential confounders. Recently, the advances in spatial transcriptomics (Ståhl *et al.* 2016) enable maintaining the spatial context of the tissue while profiling gene expressions. Its technical procedure involves capturing and sequencing RNA *in situ*, thereby preserving the spatial organization of the cellular gene expressions within the tissue architecture.

With the emergence of these novel types of data, identifying informative genes that can decipher biological mechanisms has become a keen area of interest. One key category of these genes is *spatially variable genes*. Within this scope, current works (Svensson *et al.* 2018, Shang *et al.* 2025, etc.) highlight the importance of genes in understanding spatial heterogeneity within tissues and their potential in revealing complex biological insights that traditional transcriptomics techniques cannot provide. Such a framework identifies genes that capture spatial variation and is therefore used as a crucial preprocessing step to reduce the number of genes before performing downstream tasks involving spatial variation, such as tissue segmentation (Li and Zhou 2022), cell recovery (Zhang *et al.* 2023), and resolution enhancement (Zhang *et al.* 2024). On the other hand, while these detected genes are strongly representative of spatial patterns, they are not necessarily indicative of diseases. Hence, our focus is on the second type of interesting genes, *disease-associated genes*. They are crucial in understanding the relationship between high-dimensional gene expression data and disease status, and thus informing insights for diagnosis and therapeutic strategies. Identifying disease-associated genes has been extensively studied using bulk RNA-seq data, including works by Bauer *et al.* (2008), Guala *et al.* (2014), Leiserson *et al.* (2015), and scRNA-seq data (Ruan and Wang 2021), but it is scarcely explored in spatial transcriptomics. Motivated by this, we propose a high-dimensional Bayesian model, DiSTect (DiSease DeTector), to efficiently detect the disease-associated genes accommodating for the spatial structure of transcriptomics data and can be adapted for new tissue samples, serving as a predictive tool to identify disease regions and assist in diagnosis.

DiSTect addresses three challenges imposed by the special data structure of spatial transcriptomics: high dimensionality, missing data, and computation burden.

Firstly, disease mechanisms can be driven by the simultaneous interaction of multiple genes, this requires us to consider modeling the additive effects of these genes collectively. That is, our focus will be on the conditional effects of each gene in the presence of others, which is extremely challenging due to high dimensionality. At present, dimension reduction techniques have been introduced for analyzing gene expressions (e.g. Sorzano *et al.* 2014, Feng *et al.* 2020). However, the existing methods for reducing dimensions mainly focus on a specific goal, such as extracting features for clustering, which are not general enough for identifying disease-associated genes, let alone for handling data with spatial correlations. To fill in this gap, we employed a Bayesian shrinkage model to effectively manage the association analysis in high-dimensional data.

Secondly, the presence of missing data complicates inference. In spatial transcriptomics, missing data affect both gene expression (predictors) and disease status (responses) at the same locations, meaning that if a spot is missing, neither its predictor nor its response is observed. This missing pattern often arises due to sample collection procedures or experimental errors, such as when a small section of tissue is cut out or not sampled (Lopez *et al.* 2019) while separating a slice of sample from the bulk tissue. Therefore, missingness is likely to exhibit spatial correlations, i.e. when one spot is missing, its neighboring spots are more likely to be missing, which further complicates the missing mechanism. In this article, we discuss missingness to be ignorable or nonignorable and address scenarios where the mechanism involves autocorrelation among spots.

Thirdly, spatial transcriptomics data often include a large number of spots, genes, and slices. With the decreasing costs of generating new datasets, it is crucial to ensure that the methods are scalable to large datasets. DiSTect allows users to choose the implementation methods between No-U-Turn Sampler (NUTS) (Hoffman *et al.* 2014) and variational inference, with a trade-off based on their priority between precision and computational efficiency.

**Figure 1.**
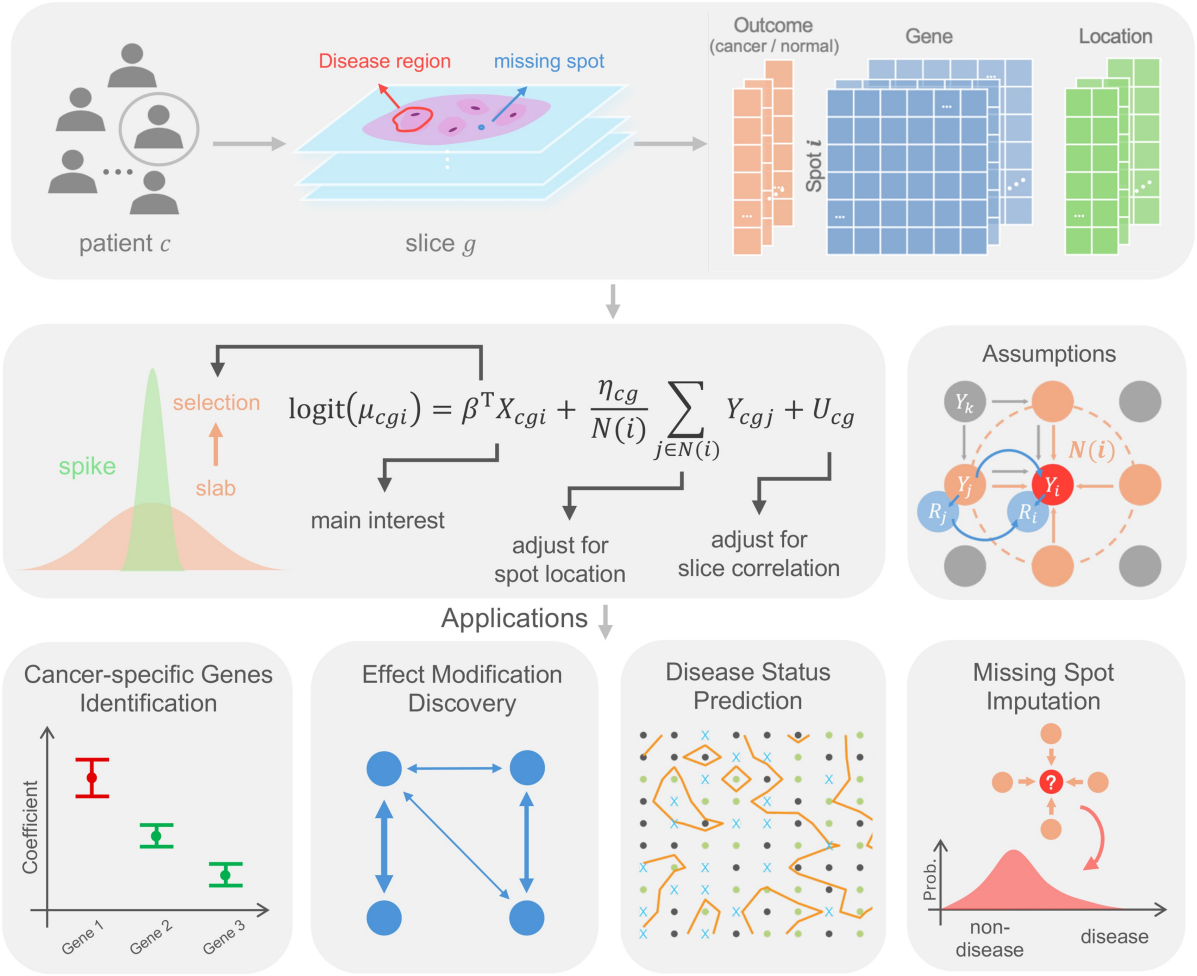
DiSTect analyzes Spatial Transcriptomics data from single or multiple tissue slices to identify genes strongly associated with disease status using a Bayesian spatial spike-and-slab model. The model explicitly accounts for within-slice spatial autocorrelation, between-slice correlation, and incorporates variable selection via a spike-and-slab prior. DiSTect facilitates four primary analytical tasks: (1) identification of disease-associated genes, (2) discovery of effect modification and regulatory structures among genes, (3) annotation of disease status for each spot in new Spatial Transcriptomics datasets, and (4) imputation of disease information for missing spots.

The detailed workflow of DiSTect is illustrated in Fig. 1. Leveraging a Bayesian spatial autologistic regression with spike-and-slab priors, DiSTect enables four downstream analyses: (i) The model identifies disease-associated genes whose spatial expression patterns are associated with disease status, supporting genetic biomarker discovery. (ii) Gene–gene interactions are examined by fitting pairwise interaction terms among selected genes, allowing exploration of potential regulatory effects relevant to disease. (iii) Posterior estimates from the model are used to predict disease status in a new unlabeled tissue. (iv) The model also imputes missing disease labels in spatial regions with unobserved measurements by leveraging local spatial context and gene expression.

## 2 Model setup

### 2.1 Main model

For i=1,…,n, let $Y_{i}$  ∈{0,1} be a binary variable to denote the disease status captured on spot *i* with a two-dimensional location coordinate $s_{i}$. For each *i*, consider *d*-dimensional covariates $X_{i}$ = ${\left(X_{i1},\ldots ,X_{id}\right)}^{⊺}$ with $X_{ik}$ denoting the expression level of gene *k* on spot *i*. Let $\mu_{i}=E(Y_{i})$. We consider the association of $Y_{i}$ and $X_{i}$ as postulated by the model:


(1) $$\text{logit}(\mu_{i})=\beta^{⊺}X_{i}+\sum_{j\neq i}\eta_{ij}Y_{j},$$

where β is the associated covariate parameter of primary interest, logit(c) = log($\frac{c}{1-c}$) and $\eta_{ij}$ are dependence parameters characterizing the correlations between the outcomes observed at different spots.

Without extra constraints, model identifiability issue arises as the number of parameters is greater than *n*. Hence, assumptions are often made to reduce the number of parameters. Here, we consider restricting the correlations with spots *i* into its neighbor set, $N(i)=\left\{j:||s_{i}-s_{j}|{|}_{2}\leq \delta \right\}$, with $s_{i}$ and $s_{j}$ being the locations of spots *i* and *j*, respectively, and δ being a pre-specified threshold. This gives a pairwise dependencies assumption,


$$P(Y_{i}|\tilde{Y_{r}},\tilde{Y_{t}})=P(Y_{i}|\tilde{Y_{r}}),$$

where $\tilde{Y_{r}}=\left\{Y_{r},r\in N(i)\right\}$, $\tilde{Y_{t}}=\left\{Y_{t},t\notin N(i)\right\}$ and the threshold δ is often taken as 1, i.e. the neighbor set is taken as the most adjacent spots. In practice, isotropic assumptions are often introduced, as suggested by Hughes *et al.* (2011), for the model presented in Equation (1). This implies that the effects of different neighboring units are assumed to be identical, reflecting the assumption that spatial correlation strength decays with distance but is directionally uniform, which is a reasonable approximation for tissues with relatively homogeneous architecture, such as germinal centers in lymphoid tissue (Oh *et al.* 2025) or undifferentiated zones within solid tumors (Du *et al.* 2023). Specifically, we define $\eta_{ij}=\frac{\eta }{\left|N(i)\right|}>0$ for i,j=1,…,n, where $\eta_{ij}$ represents the average autocorrelation factor contributed by a single neighboring unit, and |N(i)| denotes the cardinality of the neighborhood set for unit *i*, which is introduced to account for boundary effects, as detailed in Section 2.3.

Considering both pairwise dependencies and isotropic assumptions, stemming from Equation (1), a restricted autologistic model is given by:


(2) $$\text{logit}(\mu_{i})=\beta^{⊺}X_{i}+\frac{\eta }{\left|N(i)\right|}\sum_{j\in N(i)}Y_{j}.$$

We comment that in the field of spatial transcriptomics, both the pairwise dependencies and isotropic assumptions are frequently considered. For example, Zhu *et al.* (2018) considered that the spatial dependencies only depend on immediate neighboring spots. On the other hand, the proposed model (1) has the room to accommodate dependencies at greater distances by modulating the value of δ. The isotropic assumption stems from biological homogeneity, suggesting that in various tissues, the absence of a predominant direction of gene expression might be anticipated. Furthermore, with many sequencing-based gene expression profiling techniques such as 10X Visium, the resolution might not be sufficiently high enough to discern fine-grained directional patterns.

### 2.2 Joint modeling of multiple slices

In spatial transcriptomics, it is common that multiple slices from the same tumor tissue are collected and sequenced, which are often referred to as biological replicates. For instance, 10X Visium platform allows for generating spatial transcriptomic data for four serial slices (Marx 2021), enabling statistical analysis with a larger sample size. However, in such a scenario, batch effects may be the main concern and thus need to be adjusted for via statistical modeling.

In this section, we extend the model (2) to jointly model the multiple slices. We first introduce c=1,…,C to denote the index of tissue donors, where *C* is the total number of tissue donors. Then, for participant *c*, a total of $G_{c}$ slices are collected and we index each tissue slice by $g_{c}=1,\ldots ,G_{c}$. Without the loss of generality, we consider $G_{1}=G_{2}=\cdots =G_{c}=G$ and hence suppress the subscript *c* in $g_{c}$ into *g*. We further incorporate these indices into $X_{i}$ and $Y_{i}$ to indicate their source of slices, resulting in $X_{\mathrm{cgi}}$ and $Y_{\mathrm{cgi}}$, respectively. We would assume that tissues from the same participant are correlated, while tissues from different participants are independent. Based on this assumption and definition, we propose the following model,


(3) $$\text{logit}(\mu_{\mathrm{cgi}})=\beta^{⊺}X_{\mathrm{cgi}}+\frac{\eta_{cg}}{\left|N(i)\right|}\sum_{j\in N(i)}Y_{\mathrm{cgj}}+U_{cg},$$

where $U_{cg}$ is a random effect variable. We assume $U_{cg}$ follows $N(0,\Sigma_{c})$ to account for correlation among slices within an individual, where the (s,t)th element of $\Sigma_{c}$ is $\rho_{st}\sigma_{c}^{2}$. Hence, the covariance matrix $\Sigma_{c}$ can be fully parameterized. Such a specification implicitly assumes $U_{c_{1}g_{1}}$ and $U_{c_{2}g_{2}}$ are independent for any $\left(g_{1},g_{2}\right)$ of $c_{1}\neq c_{2}$.

To account for the nature of correlation structure among slices from the same donor, we consider an exchangeable structure assuming that the pairwise correlations are the same, i.e. for ∀s≠t, $\rho_{st}=\rho$ and for ∀s=t, $\rho_{st}=1$. This assumption makes sense when their processing pipelines are often standardized. This consistency allows for the assumption of consistent spatial effects, leading to the constraint that $\eta_{cg}=\eta$ for each *c* and *g*. Consequently, it is natural to consider the correlation between two adjacent slices influenced by their relative positions. Under such circumstances, an autoregressive correlation structure would be more appropriate, where $\rho_{st}=\rho^{\left|g_{s}-g_{t}\right|}$ for slices *s* and *t*. This option is implemented in the software package and can be specified by the user based on the biological context.

### 2.3 Boundary effect and missing spots

Model (1) assumes all $Y_{j}$ to be observed. However, $Y_{j}$ can be unknown under two scenarios. The first one occurs for a spot (e.g. Spot *i*) on the outer edges. In this case, its neighboring spot (e.g. Spot *j*) may be unobserved because of the natural boundary of the tissue. Consequently, the spots on the boundary will have fewer number of neighbors than others. Therefore, instead of just using a single parameter to account for autocorrelations $\eta_{ij}=\eta$ (e.g. Hughes *et al.* 2011), we adjust it by the number of neighbors in Equation (2).

On the other hand, for the missing spots isolated in the interior of the tissue, their missingness often results from sample collection procedure. This missingness cannot be ignored because the corresponding $\eta_{ij}$ might be non-zero. Without proper adjustment, ignoring them will lead parameter estimation of Equation (1) to be biased, as it is equivalent to forcing $\eta_{ij}$ to be 0. In order to address this, we introduce new notations, $Y_{j}^{\mathrm{obs}}$ and $Y_{j}^{\mathrm{mis}}$, respectively, to distinguish the observed and unobserved disease status for spot *j*, and a binary variable $R_{j}$ to indicate the observation status of $Y_{j}$. Then, model (2) can be rewritten as:


(4) $$\text{logit}(\mu_{i})=\beta^{⊺}X_{i}+\frac{\eta }{N(i)}\sum_{j\in N(i)}\left\{Y_{j}^{\mathrm{obs}}R_{j}+Y_{j}{\;}^{\mathrm{mis}}(1-R_{j})\right\},$$

where $R_{j}$ is 1 if $Y_{j}$ is observed, and otherwise, it would be 0.

Because biological tissue is naturally continuous and interconnected, the failure of collecting one spot during the slice-collection procedure might increase the chance of the adjacent spot being missed as well, leading to the missing pattern tending to be autocorrelated. Hence, we consider an autocorrelated missing pattern:


Ri /⊥Rj and Ri⊥Rk|Rj  for  k∉N(i) and j∈N(i).

However, such a complex missing pattern might be *ignorable* under certain circumstances. For example, the missing spots are often due to errors in the experimental operations such as tissue slicing, where the missingness is independent of the data observed on the tissue. Under this assumption, we consider the missing mechanism to be *missing completely at random* (MCAR). That is to say, for i=1,…,n, $P(R_{i}|\tilde{X},\tilde{Y})=P(R_{i})$, where $\tilde{X}={\left(X_{1}^{⊺},\ldots ,X_{n}^{⊺}\right)}^{⊺}$ and $Y={\left(Y_{1},\ldots ,Y_{n}\right)}^{⊺}$.

On the other hand, the experimenter’s bias can influence the bias pattern, leading to the missing occurring differently according to the disease outcome. For example, the experimenter might have less incentive to include the normal regions and therefore process them with less care, causing the control region to have a different missing rate compared to the disease region. In this scenario, the ignorable assumption may be no longer reasonable. Instead, we model the missing status of spot *i* by its outcome as well as its neighboring missing status:


(5) $$\text{logit}\;P(R_{i}=1)=\gamma_{0}+\gamma_{1}Y_{i}+\frac{\gamma_{2}}{\left|N(i)\right|}\sum_{j\in N(i)}R_{j},$$

where $\gamma ={\left(\gamma_{0},\gamma_{1},\gamma_{2}\right)}^{⊺}$ are associated parameters. The values of $\gamma_{1}$ and $\gamma_{2}$ determine the complexity of the missingness mechanism. When $\gamma_{2}\neq$ 0, the missing mechanism is subject to spatial autocorrelation. However, when $\gamma_{1}=0,$ the missingness degenerates into an ignorable form, making model (5) unnecessary and $\gamma_{2}$ to become irrelevant.

Furthermore, although in Equation (5) only $Y_{i}$ is explicitly included in the missingness model, regional disease information of surrounding spot $Y_{j}$ is indirectly incorporated through two pathways: (i) $R_{i}$ depends on neighboring $R_{j}$, each of which depends on $Y_{j}$ via model (5), forming the path $R_{i}↔R_{j}\leftarrow Y_{j}$; and (ii) since $R_{i}$ depends on $Y_{i}$ and $Y_{i}$ is spatially correlated with neighboring $Y_{j}$, we also have $R_{i}\leftarrow Y_{i}↔Y_{j}$. Together, these dependencies allow the model to reflect spatially structured missingness without explicitly including $\sum_{j}Y_{j}$, and thus avoiding redundancy and identifiability issues. A visual illustration of these dependency paths is provided in Fig. 1 (middle right).

## 3 Methodology

### 3.1 Spike and slab autologistic model

To fulfill variable reduction, we integrate spike and slab priors with spike component specified as absolutely continuous spike into the following Bayesian hierarchical models:


(6) $$\begin{array}{c} (Y_{i}|X_{i})\overset{i.i.d}{∼}\text{Bernouli}(\mu_{i}),i=1,\ldots ,n,\\ \eta ∼\text{Uniform}(0,c_{1}),\\ (\beta_{k}|\iota_{k},\tau_{k}^{2})\overset{i.i.d}{∼}\text{Normal}(0,\iota_{k}\tau_{k}^{2}),\\ (\iota_{k}|v_{0},w)\overset{i.i.d}{∼}(1-w)\delta_{v_{0}}(\cdot )+w\delta_{1}(\cdot ),\\ (\tau_{k}{\;}^{–2}|b_{1},b_{2})\overset{i.i.d}{∼}\text{Gamma}(b_{1},b_{2}),k=1,\ldots ,d,\\ w∼\text{Uniform}[0,1], \end{array}$$

where $\mu_{i}$ is modeled by Equation (1), $c_{1}$, $v_{0}$, $b_{1}$, $b_{2}$ are hyperparameters to be prespecified, $\delta_{v}(\cdot )$ function is a discrete measure concentrated at value *v*, and the choice for $v_{0}$ is usually a small value near zero. We comment that this framework is also called *Normal Mixture of Inverse Gamma* (NMIG) proposed by Ishwaran and Rao (2005), which takes full advantage of the conjugate Inverse Gamma prior for $\tau_{k}^{2}$ and leads to highly efficient computation when applied to high-dimensional datasets.

### 3.2 Model with multiple tissue slices available

To accommodate the scenarios where datasets are collected with multiple replicates of slices from different individuals, as considered in Section 2.2, we extend the model (6). The data-generating process for $Y_{i}|X_{i}$ is now indexed by *c*, *g*, and *i*:


$$Y_{\mathrm{cgi}}|X_{\mathrm{cgi}}\overset{i.i.d}{∼}\text{Bernouli}(\mu_{\mathrm{cgi}}),$$

for c=1,…,C,g=1,…,G,i=1,…,n, where $\mu_{\mathrm{cgi}}$ is modeled by Equation (3). Since Model (3) introduces a latent random effect $U_{cg}$, here, we further extend Equation (6) into a Bayesian hierarchical model by including $U_{cg}∼\text{MVN}(0,\Sigma_{c}),$ where $\sigma^{2}$ in $\Sigma_{c}$ are assigned with priors


$$\begin{array}{c} \sigma_{c}^{–2}\overset{i.i.d}{∼}\text{Gamma}\;(b_{3},b_{4}),\;\text{for}\;c=1,\ldots ,C,\;\text{and}\\ \rho ∼\text{Uniform}(b_{5},b_{6}). \end{array}$$

Here, the parameters denoted as $b_{3}$, $b_{4}$, $b_{5}$, $b_{6}$ are characterized as hyperparameters to be specified.

### 3.3 Bayesian inference

Inference about the parameter $\theta ={\left(\eta ,\beta ,\iota ,\tau ,w\right)}^{⊺}$ for Section 3.1 or $\theta ={\left(\eta_{cg},\beta ,\iota ,\tau ,w,U_{cg},\sigma_{c},\rho \right)}^{⊺}$ are carried out by the following posterior distribution:


(7) $$f(\theta |y,x)=\frac{f(\theta ,y|x)}{f(y|x)}\propto f(y|x,\theta )\pi (\theta ),$$

where f(θ,y|x) is the joint distribution of *Y* and θ conditional on *X*, π(θ) is the prior distribution of parameter θ.

## 4 Inference with missing data

With the presence of missing data, the inference of the θ starts with the joint distribution of (Y,R), given by:


(8) f(y,r|x,θ,γ)=f(y|x,θ)f(r|y,γ).

Then, the inference based on the observed data is given by:


(9) $$f(y^{\mathrm{obs}},r|x,\theta ,\gamma )=\sum_{y^{\mathrm{mis}}}f(y,r|x,\theta ,\gamma ).$$

When the data is *missing completely at random* (MCAR), which means that the missing data mechanism *R* is completely independent of *Y*, Equation (8) can be further factorized as


f(y,r|x,θ,γ)=f(y|x,θ)f(r|γ).

This indicates that the likelihood f(y|x,θ) is not influenced by the likelihood of f(r|γ), and hence we can ignore the model of *R* when conducting the inference of θ and carry out the inference only via f(y|x,θ). Here, Equation (9) would become:


(10) $$f(y^{\mathrm{obs}}|x,\theta ,\gamma )=\sum_{y^{\mathrm{mis}}}f(y|x,\theta ,\gamma ).$$

The inference on (10) involves a summation over all potential binary configurations of $y^{\mathrm{mis}}$, which can be computationally expensive when the number of missing spots is substantial. Therefore, we incorporate a data augmentation procedure by treating $y^{\mathrm{mis}}$ as a latent variable and imputing them via iterative multiple imputation with a Gibbs sampler. This is equivalent to adjusting Equation (6) by considering the separated data-generating process of outcome $Y_{i}$ according to whether spot *i* is observed. Specifically, let M denote the index set of spots are missing, for i=1,…,n, j∈N(i), and k∈M,


(11) $$\begin{array}{c} Y_{k}^{\text{mis}}|Y_{w}^{\text{obs}},Y_{w}^{\text{mis}},R_{w}∼\text{Bernoulli}\left(\mu_{k}^{*}\right),\\ Y_{i}^{\text{obs}}|X_{i},Y_{j}^{\text{obs}},Y_{j}^{\text{mis}\;},R_{j}\;\overset{i.i.d.\;}{∼}\text{Bernoulli}(\mu_{i}), \end{array}$$

where $\mu_{k}^{*}=\frac{1}{\left|N(k)\right|}\sum_{w\in N(k)}\left\{Y_{w}^{\text{obs}}R_{w}+Y_{w}^{\text{mis}}(1-R_{w})\right\}$, $\mu_{i}$ follows Equation (4) and |N(i)| is the cardinality of N(i). As we do not observe $X_{k}$ for k∈M, here $Y_{k}^{\mathrm{mis}}$ is imputed according to the proportion of disease spots in the neighboring spots of *k*.

When missing is nonignorable, we carry out inference on Equation (9) directly. To facilitate this, we extend (11) to incorporate the missing mechanism of $R_{i}$ via Equations (4) and (5), which gives, for i=1,…,n, j∈N(i), and k∈M,


$$\begin{array}{c} Y_{i}^{\text{obs}}|X_{i},Y_{j}^{\text{obs}},Y_{j}^{\text{mis}\;},R_{j}\overset{i.i.d.}{∼}\text{Bernoulli}(\mu_{i}),\\ Y_{k}^{\text{mis}}∼\text{Bernoulli}\left(\mu_{k}^{*}\right),\\ R_{i}|Y_{i},R_{j}∼\text{Bernoulli}\left\{P(R_{i}=1)\right\}, \end{array}$$

where $\mu_{k}^{*}$ is as in (11) and $P(R_{i}=1)$ is specified in Equation (5).

To facilitate parameter estimation, DiSTect offers two implementation options: No-U-Turn Sampling (NUTS) and Automatic Differentiation Variational Inference (ADVI). Detailed descriptions of the implementation procedures are provided in Section A1, available as supplementary data at *Bioinformatics* online.

## 5 Downstream analyses

After obtaining posterior estimates of parameters from DiSTect, one can conduct four primary downstream analyses, each addressing specific biological and analytical goals:

- **Disease-specific gene identification.** Disease-specific genes can be identified based on the magnitude and significance of their regression coefficients β. Specifically, we rank genes according to standardized effect sizes, defined as the ratio of each gene’s posterior mean coefficient to its posterior SD (analogous to a *Z*-score). Genes exhibiting large standardized effect sizes were selected, as these indicate strong associations with disease status, enabling targeted biological interpretation and biomarker discovery.
- **Gene–gene interaction discovery.** To investigate potential regulatory, we consider pairwise interaction terms among the top-ranked genes selected in the previous step. Specifically, we extend our original logistic regression model in Equation (2) by adding interaction terms $X_{ik}X_{il}$ for gene pairs (k,l) among selected genes:
  (12) $$\text{logit}(\mu_{i})=\beta^{⊺}X_{i}+\sum_{\left(k,l\right)}\beta_{kl}^{\text{int}\;}X_{ik}X_{il}+\frac{\eta }{\left|N(i)\right|}\sum_{j\in N(i)}Y_{j}.$$
    We estimate these interaction parameters $\beta_{kl}^{\text{int}}$ and assess their statistical significance and biological relevance by calculating standardized effect sizes, defined as the posterior mean divided by the posterior SD. Interaction terms with standardized effect sizes exceeding a threshold of 1.96 are retained, indicating the interactions are worthy of further biological exploration.
- **Spatial disease prediction.** DiSTect supports predictive annotation of disease status for tissue spots without known labels. Specifically, posterior parameter estimates ($\hat{\beta },\hat{\eta }$) from previously analyzed slices or patients are used to compute predictive probabilities of disease status for new unlabeled tissue spots from Equation (2). The uncertainty of the prediction can be quantified using the posterior samples.
- **Missing disease status imputation.** Finally, DiSTect probabilistically imputes missing disease labels arising from technical artifacts or sample preparation issues. For any spot *i* with missing disease status, posterior predictive distributions conditioned on neighboring observations are used to estimate the probability of disease presence following Equation (5).

## 6 Simulation studies

We conduct simulation studies to assess the performance of the proposed model in parameter estimation. We perform three simulation studies to compare the autologistic model (2) with the naive approach, where the spatial patterns are disregarded. In Simulation 1, we consider all the spots are displayed on a single slice; in Simulation 2, we consider that a more complex data structure with the spots may come from different slices; and Simulation 3 includes the situation where missing data are present. Detailed descriptions of each simulation can be found in Sections A2.1–A2.3, available as supplementary data at *Bioinformatics* online.

In Simulation 1, the naive model generally exhibits larger bias in parameter estimates. In contrast, DiSTect adjusts for spatial effects and consistently achieves lower average bias across various levels of spatial correlation (Figs B3 and B4 and Tables C1–C3, available as supplementary data at *Bioinformatics* online). Notably, the NUTS-based implementation of our model yields smaller biases compared to the ADVI-based approach. Both methods provide reasonable posterior SD estimates and maintain valid coverage probabilities. However, the variational inference approach (ADVI) offers substantially improved computational efficiency, as reflected in reduced computation time (Fig. B2, available as supplementary data at *Bioinformatics* online). We conduct a sensitivity analysis to assess the impact of misspecifying the parameter δ, which governs the neighborhood size. The results indicate that employing a relatively smaller neighborhood radius leads to robust model performance despite the misspecification (Fig. B5, available as supplementary data at *Bioinformatics* online). In addition, a separate sensitivity analysis is performed to evaluate the model’s performance under violations of the isotropic assumption. The findings suggest that the estimation of β remains comparable even when isotropy is not satisfied (Fig. B6, available as supplementary data at *Bioinformatics* online). Simulation 2 demonstrates that our model performs robustly in both point and variance estimation across varying degrees of heterogeneity and spatial correlation among slices (Fig. B7 and Table C4, available as supplementary data at *Bioinformatics* online). We additionally perform a sensitivity analysis to evaluate the impact of the misspecification in cross-tissue correlation. It is shown that the misspecification mainly affects the efficiency of the estimators, not the point estimates (Fig. B8, available as supplementary data at *Bioinformatics* online). In Simulation 3, we assess the performance of DiSTect in the presence of missing data. Our model continues to yield valid inference and substantially lower estimation bias compared to the naive model, under both ignorable and non-ignorable missing data mechanisms (Figs B9 and B10 and Tables C5 and C6, available as supplementary data at *Bioinformatics* online).

**Figure 2.**
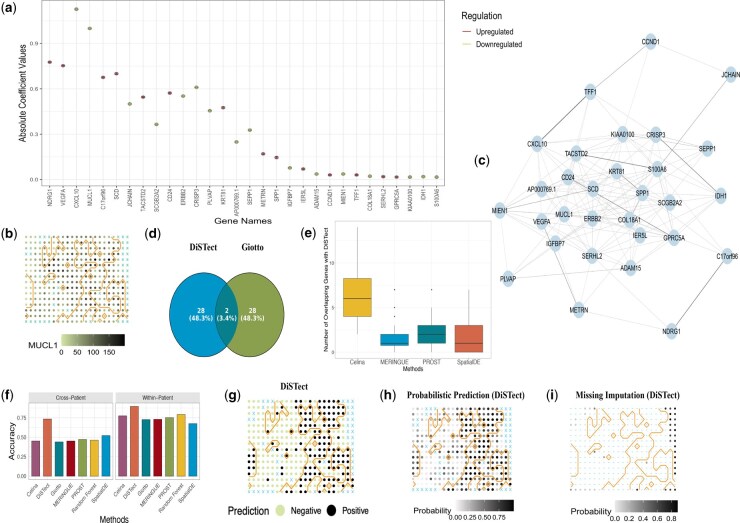
Disease-associated genes, interactions, overlap with baselines, and DiSTect outputs. (a) Posterior absolute coefficient values ($\left|\hat{\beta }\right|$) with 95% credible intervals for the 30 top-ranked genes; color encodes direction of association (red, upregulated; green, downregulated). (b) Spatial expression of *MUCL1*; darker dots indicate higher normalized expression. The orange contour delineates the ground-truth boundary between cancer and non-cancer regions; blue x marks missing spots. (c) Pairwise interaction network among the top 30 genes. An edge is drawn when the standardized interaction effect (posterior mean/posterior SD) exceeds 1.96; edge width is proportional to effect magnitude. (d) The overlap genes between DiSTect and Giotto. (e) Overlap counts between DiSTect-selected genes and SVGs from Celina, MERINGUE, PROST, and SpatialDE (per-slice, top-30 comparison). (f) Prediction accuracy under cross-patient (leave-one-patient-out) and within-patient (3-fold) evaluations across methods. (g) Thresholded disease predictions from DiSTect (probability >0.5); black, predicted cancer; green, predicted non-cancer. Orange contour and blue x as in (b). (h) Posterior predictive probabilities ${\hat{\mu }}_{i}$ for each spot from DiSTect; grayscale indicates P(disease). (i) Imputation for missing spots: posterior P(disease) is shown at missing locations only; observed spots are indicated by blue “–” symbols.

## 7 Analysis of HER2+ breast cancer spatial transcriptomic data

We illustrate the usage of the proposed method, by applying it to HER2+ breast cancer spatial transcriptomic data (Andersson *et al.* 2021). The datasets include gene expression measurements for over 15 000 genes from eight individuals (labeled with patient ID A–H). Among them, patients A–D have six slices, and patients E–H have three. In each slice, an average of 346 spots is sampled. The disease statuses are manually annotated by the pathologists, and each sampled spot is labeled as either cancer or non-cancer. Our objectives in the study are (i) to identify the genes that are specific to the disease using the proposed model; and (ii) then construct the predictive model to inform the risk of cancer status on each spot for any newly collected spatial transcriptomic sample.

Before analysis, we first perform preprocessing procedures. A gene filtering procedure is conducted to exclude the genes whose total counts are below 300. As the number of filtered genes are different for each slice, we take a common subset to ensure that only mutual genes are kept for the slices from the same patient. The gene expressions, originally recorded as counts (e.g. denoted as *X*), undergo a log(X+1) transformation and are converted into a continuous scale. We introduce $Y_{i}$ to be a binary variable to denote the outcome according to its disease status, where “0” or “1” stand for whether spot *i* is located in a non-cancer or cancer region, respectively. Model (3) is used to analyze multiple slices jointly. To accommodate for missingness, we consider the missing mechanism to be ignorable because the major cause of the missingness in this dataset is the tissue-slicing procedure, and no experimental biases are observed or recorded. Hence, we apply the strategy for handling ignorable missing in Section 2.3. The specification of hyperparameters is displayed in Table C7, available as supplementary data at *Bioinformatics* online. The method is implemented by ADVI with a total computation time amounted to 25 h and 32 min on a system with an M3 chip and 24 GB memory.

DiSTect identifies the 30 genes that demonstrate the highest levels of effect-to-standardized-deviation ratio (Fig. 2a). To closely examine how the expressions of these genes are associated with the spatial pattern of both cancer and non-cancer regions within the tissue, spatial visualization of gene expressions is generated in Fig. 2b and Fig. B11ab, available as supplementary data at *Bioinformatics* online. For example, the expression level of *MCUL1* ($\hat{\beta }=-1.020$, 95% CrI = [−1.024,−1.016]) is elevated mainly in non-cancer regions and rarely in cancer regions, suggesting its potential role in inhibiting cancer development. The finding is consistent with the results in Fig. 2a that the estimated coefficient for the *MUCL1* gene is negative. Similarly, as depicted in Fig. B11ab, available as supplementary data at *Bioinformatics* online, the estimated coefficients for the *NDRG1* ($\hat{\beta }=0.777$, 95% CrI = [0.774, 0.780]) and *VEGFA* ($\hat{\beta }=0.752$, 95% CrI = [0.749, 0.756]) genes are positive, indicating a likely promotion of cancer development. Interestingly, despite this, the elevated expression locations for these two genes differ, suggesting distinct roles in cancer progression. These findings further support the necessity of modeling disease status by considering multiple gene expressions jointly. In addition, the estimated η is 2.936, 95% CrI=[2.926, 2.946], representing a notable level of spatial autocorrelation among adjacent spots.

Motivated by these findings, we further investigate whether gene–gene interactions are essential in the cancer development process. We use model (12) by adding the two-way interactions among the selected 30 genes to investigate their effects on cancer. Then, we select the interaction terms with an effect-to-standardized-deviation ratio >1.96 (Fig. 2c). Numerous gene–gene interactions are strongly indicative of the disease status. Notably, we identify several hub genes (e.g. *SCD* and *ERBB2*) according to their extensive correlations within many other genes. In addition, *VEGFA* is known to be related to the proliferation and migration of vascular endothelial cells, which secure a blood supply for growth and are hence upregulated in tumor tissues (Qin *et al.* 2023). Through this gene–gene interaction network, we identify several genes, such as *CXCL10* ($\hat{\beta }=-1.0908$, 95% CrI = [−1.0948,−1.0867]) and *MIEN1* ($\hat{\beta }=-0.0326$, 95% CrI = [−0.0367,−0.0285]), that potentially interact with *VEGFA* in cancer progression.

We compare DiSTect with existing approaches for identifying differentially expressed genes (DEGs), such as Giotto (Dries *et al.* 2021), as well as methods for detecting spatially variable genes (SVGs), including SpatialDE (Svensson *et al.* 2018), Celina (Shang *et al.* 2025), PROST (Liang *et al.* 2024), and MERINGUE (Miller *et al.* 2021). We first examine the overlap between the genes identified by DiSTect and those selected by benchmark methods. For DEGs detected using Giotto, only two genes overlapped with DiSTect, while the majority of genes are unique to each method (Fig. 2d). For SVG methods, since they do not support multi-slice analysis, we apply them to individual slices and select the top 30 genes per slice. Across methods, the overlap with DiSTect-selected genes is generally low (Fig. 2e), highlighting substantial differences in gene prioritization. These findings suggest that by explicitly accounting for conditional dependence and disease context, DiSTect provides complementary information to existing approaches for gene discovery. We further evaluate predictive performance using cross-validation at both within-patient and cross-patient levels. The within-patient analysis uses a 3-fold cross-validation per individual, while the cross-patient setting follows a leave-one-patient-out strategy. For each method, we select the top 30 ranked genes to fit a two-way interaction model via DiSTect and perform prediction. For SVG-based methods, the 30 genes are selected from only one randomly chosen slice within the training dataset due to the lack of support for multi-slice analysis, while for other methods the 30 genes are identified from the entire set of training data. In addition, we also compare the results to a random forest trained on all available genes. DiSTect consistently outperform all competing methods in both evaluation settings (Fig. 2f), with representative deterministic prediction exhibited in Fig. 2g and Fig. B11(d–h), available as supplementary data at *Bioinformatics* online in the Materials.

In addition to evaluating methods in decision-making as reflected by the deterministic predictions, we also investigate the uncertainty of predictions across the entire tissue by evaluating the predicted $\hat{\mu }$ at each spot. Typically, a prediction is more uncertain when the predicted probability $\hat{\mu }$ is closer to 0.5. Conversely, the prediction is more confident. As illustrated in Fig. 2h, while the prediction is generally consistent with the ground truth annotations, a strong heterogeneity in prediction confidence is observed across the tissue. In particular, spots around the boundary between the cancer region and the non-cancer region tend to exhibit a greater uncertainty. This is expected because the transition zone between different tissue types often contains cells with mixed morphological and molecular characteristics, leading to higher ambiguity in classification. Cancer cells can infiltrate and intermingle with normal cells, creating changes that complicate the distinction between regions, thus increasing prediction uncertainty (Scimeca *et al.* 2014).

Lastly, we explore how DiSTect can utilize information from gene expression patterns to impute unknown disease status for missing spots in spatial transcriptomics. As an example, for patient A slice 2, DiSTect generates posterior probabilities indicating the likelihood that each missing spot belongs to the disease group. The probabilistic imputation results are presented in Fig. 2i. These imputed disease statuses demonstrate strong spatial coherence with the surrounding observed spots.

###  Generalization across diseases and platforms

To assess generalizability beyond HER2+ breast cancer and 10X Visium, we evaluate DiSTect on a STARmap PLUS dataset from the mouse brain with Alzheimer’s pathology (Zeng *et al.* 2023). We focus on two 13-month disease replicates and defined disease labels by proximity to amyloid-β plaques (within 20μm) and set a neighborhood with a 60-pixel radius (approximately four neighbors as in 10X Visium). In Replicate 1, DiSTect identifies eight disease-associated genes, with the top signals (*Cst7*, *Trem2*, *C1qa*, *Gfap*) concordant with prior findings (Zeng *et al.* 2023). We also detect a gene–gene interaction consistent with microglial activation, where *Hexb* modulated the effect of *Trem2*. For prediction on Replicate 2, DiSTect achieves 97.1% accuracy, compared with 96.8% using DEGs selected by Giotto; a model based on MERINGUE SVGs produces no positive calls in this dataset. Full analyses, figures, and implementation details are provided in Section A3, Fig. B12, available as supplementary data at *Bioinformatics* online.

## 8 Discussion

In this article, we introduce a Bayesian shrinkage spatial model that incorporates a spike-and-slab prior to identify the disease-specific genes and construct predictive models to inform cancer diagnosis. The shrinkage prior of the Bayesian spatial model enables the selection of the key relationships by shrinking negligible coefficients to zero. This interpretable approach facilitates both the selection of relevant genes for the disease and the prediction on new data. Additionally, we extend our model to address missing data and model multiple slices simultaneously, thereby enhancing its applicability to various settings of cancer genomics studies and enabling the potential of multi-site analysis.

It is well recognized that spatial transcriptomics platforms differ in spatial resolution, raising the question of whether the neighborhood size parameter δ should be adapted accordingly. We recommend a conservative default of δ=1 for lower-resolution platforms such as 10X Visium, and for single-cell-resolution data (e.g. MERFISH or Xenium), a choice that includes approximately 30–50 cells per neighborhood to mimic the effective coverage of δ=1 in lower-resolution settings. Sensitivity analysis is advised for tissues with broader spatial autocorrelation. To our knowledge, no systematic study has examined the relationship between platform resolution and optimal neighborhood size for spatial modeling, and this remains an open area for further investigation. In addition, while our framework captures regional dependence indirectly, explicitly modeling $R_{i}$ as a function of aggregated neighboring outcomes (e.g. $\sum_{j\in N(i)}Y_{j}$) could better address region-level, disease-dependent missingness and is a promising extension. Finally, DiSTect currently assumes exchangeable $\rho_{st}$ and isotropic $\eta_{ij}$, which are reasonable for relatively homogeneous tissue regions but may be less appropriate in heterogeneous microenvironments with strong directional structures. Allowing $\eta_{ij}$ to vary spatially or incorporating directional kernels could better capture such anisotropy in future implementations.

Our evaluation relies on within-patient cross-validation and leave-one-patient-out testing, which quantify internal generalization but not external transfer. When suitably matched external datasets become available, ideally with the same platform, tissue context, and comparable processing by independent experimenters, DiSTect can be benchmarked to assess out-of-cohort performance.

## Supplementary Material

btaf530_Supplementary_Data

## Data Availability

All codes and data involved in this paper are available on Github (https://github.com/StaGill/DiSTect).
