# Supplementary material for: DiSTect: a Bayesian model for disease-associated gene discovery and prediction in spatial transcriptomics
Source: Bioinformatics. 2025 Sep 22;41(10):btaf530. doi: 10.1093/bioinformatics/btaf530 (PMC12502917; doi:10.1093/bioinformatics/btaf530)
Supplement: btaf530_Supplementary_Data [file btaf530_supplementary_data.pdf]

August 23, 2025

Supplementary Materials for "DiSTect: a Bayesian spatial model for disease-associated gene discovery and prediction in spatial transcriptomics"

Qicheng Zhao, Anji Deng, and Qihuang Zhang[1]

Keywords: Bayesian Model, Disease-Specific Gene, Missing Data, Spatial Transcriptomics, Statistical Genomics, Variable Selection

Short title: DiSTect: Bayesian Model for Disease-Specific Gene Detection

[1]Corresponding Author: Department of Epidemiology, Biostatistics and Occupational Health, McGill University, Montreal, Quebec, Canada H3A 1G1, qihuang.zhang@mcgill.ca

A1 Parameter Estimation Procedure

A1.1 No-U-Turn Sampling

We adopt the No-U-Turn Sampler (NUTS, Hoffman et al., 2014), a modified Hamiltonian Monte Carlo (HMC) algorithm commonly used for parameter estimation in Bayesian hierarchical model, particularly for absolutely continuous spike prior in Model (6) in main text.

To describe the sampling algorithm, we first let θ_t be the parameters generated at the tth state of the Markov chain from our target distribution of θ, and let p_t to be the auxiliary Gaussian momentum variables generated at the tth step, which is assumed to follow the distribution $N(0, M)$ independent of t and θ, with M being a covariance matrix prespecified. We further define $H(\theta_t, p_t) = -\log f(\theta_t) + \frac{1}{2} p_t^\intercal M^{-1} p_t$ with θ_t and p_t independent for every t. The joint probability density function of θ_t and p_t can be derived as

$$f(\theta_t, p_t) = \exp\{\log f(\theta_t) + \log f(p_t)\} \propto \exp\left\{\log f(\theta_t) - \frac{1}{2} p_t^\intercal M^{-1} p_t\right\}$$
$$\propto \exp\{-H(\theta_t, p_t)\},$$

where $H(\theta_t, p_t)$ is often known as Hamiltonian equation, and $f(\theta_t)$ is the probability density function of θ_t. Then, θ_t is sampled by the NUTS algorithm via a three-step procedure.

- **Step 1**: given initial values of θ_{t-1} and p_{t-1}, we apply slice sampling (Neal, 2003) by generating an auxiliary variable u_t with

$$p(u_t|\theta_{t-1}, p_{t-1}) \sim \text{Uniform}[0, \exp\{-H(\theta_{t-1}, p_{t-1})\}], \tag{1}$$

  such that the joint distribution of $u_t, \theta_{t-1}, p_{t-1}$ is given by

$$f(u_t, \theta_{t-1}, p_{t-1}) = \begin{cases} \frac{1}{C}, & 0 \le u_t \le \exp\{-H(\theta_{t-1}, p_{t-1})\}, \\ 0, & \text{otherwise}, \end{cases}$$

  where $C = \int \exp\{-H(\theta_{t-1}, p_{t-1})\} d\theta_{t-1} dp_{t-1}$ is a normalizing constant.

- **Step 2**: the sampling of (θ_t, p_t) is then driven by sampling $(u_t, \theta_{t-1}, p_{t-1})$ alternatively from the joint density distribution $f(u_t, \theta_{t-1}, p_{t-1})$.

  For a u_t obtained from Step 1, we adopt doubling method (Neal, 2003) to sample a series of candidates, which are B_t=$\{(\theta^{*1}, p^{*1}), \ldots, (\theta^{*m}, p^{*m})\}$ from

$$(\theta^{*i}, p^{*i}) \in S = \left\{u_t \leq \exp(-H(\theta^{*i}, p^{*i}))\right\}, \text{for } i \in \{1, \ldots, m\}.$$

  With invariant property of $H(\theta, p)$ (Neal et al., 2011), the doubling method proceeds randomly taking either forward or backward leapfrog formula to sample (θ^{*i}, p^{*i}), for $i = 1, \ldots, m$. Specifically, we start with initial values $\tau = 0, i = 0$, and $\theta(\tau) = \theta_{t-1}$. Then we iteratively:

  1. generate momentum $p(\tau) = p_t$ from the distribution $N(0, M)$;
  2. generate v_i uniformly from $\{-1, 1\}$;
  3. conduct a half step for p_t with
$$p(\tau + \frac{1}{2}v_i\epsilon) = p(\tau) - \frac{1}{2}v_i\epsilon\frac{\partial f(\theta)}{\partial\theta}(\theta(\tau)), \tag{2}$$
     where ϵ is the integration step size pre-determined;
  4. perform the second half step for θ and p respectively,
$$\theta(\tau + v_i\epsilon) = \theta(\tau) + v_i\epsilon p(\tau + \frac{1}{2}v_i\epsilon), \tag{3}$$
     and
$$p(\tau + v_i\epsilon) = p(\tau + \frac{1}{2}v_i\epsilon) - \frac{1}{2}v_i\epsilon\frac{\partial f(\theta)}{\partial\theta}\left\{\theta(\tau + v_i\epsilon)\right\}; \tag{4}$$
  5. the above (2), (3), and (4) complete one full leapfrog step for θ and p, and we assign $\theta^{*i} = \theta(\tau + v_i\epsilon)$,and $p^{*i} = p(\tau + v_i\epsilon)$;
  6. repeat steps 2) - 5) for 2^i times;
  7. iterate $i = i + 1$, doubling proceeds will stop when
$$(\theta^+ - \theta^-)^\intercal p^- < 0 \text{ or } (\theta^- - \theta^+)^\intercal p^+ < 0,$$
     where (θ^+, p^+) and (θ^-, p^-) are respectively $(\max_{j\leq i}\theta^{*j}, \max_{j\leq i}p^{*j})$, $(\min_{j\leq i}\theta^{*j}, \min_{j\leq i}p^{*j})$.

uniformly sample (θ^*, p^*) from B_t and accept it as (θ_t, p_t)=(θ^*, p^*).

In practice, NUTS can be implemented by package **Rstan** in R.

A1.2 Automatic Differentiation Variational Inference

While the NUTS algorithm efficiently samples the parameters from their posterior distributions, its scalability remains a challenge, especially in the context of large-scale and high-dimensional datasets as commonly encountered in spatial transcriptomics. To address the high computational demands of our model, we adopt Automatic Differentiation Variational Inference (ADVI), which was proposed by Kucukelbir et al. (2017). This method serves as a more computationally efficient alternative by approximating the posterior distribution of the parameters, taking the tradeoff between computational feasibility and the accuracy of the estimated parameters.

A1.2.1 Transformation of constrained variables

ADVI requires the support of each parameter to be $\mathbb{R}$. To facilitate this, we first map the support of $\theta = (\eta, \beta, \iota, \tau, w)^\intercal$ into $\mathbb{R}^{p_\theta}$, where p_θ is the dimension of θ, by taking the transformations $T(\theta) = (\Phi^{-1}(\frac{\eta}{c_1}), \beta, log(\tau^2), \Phi^{-1}(w))$, and let the resulting transformed parameters be $\zeta = T(\theta)$. The transformed joint density $f(\zeta, y|x)$ has the representation, $f(\zeta, y|x) = f(T^{-1}(\zeta), y|x)|\det\ J_{T^{-1}}(\zeta)| = f(y|x, T^{-1}(\zeta))f(T^{-1}(\zeta))|\det\ J_{T^{-1}}(\zeta)|$, where the detailed form of $f(\zeta, y|x)$ is given by

$$
\begin{aligned}
f(\zeta, y \mid x) = \prod_{i=1}^{n} \mu_i^{y_i}(1-\mu_i)^{1-y_i} \Bigg\{ & (1-\Phi(\zeta_4)) \times \frac{1}{\sqrt{2\pi v_0 e^{\zeta_3}}} \exp\left(-\frac{\beta^2}{2v_0 e^{\zeta_3}}\right) \\
& \times \frac{b_2^{b_1}}{\Gamma(b_1)} e^{\zeta_3(-b_1-1)} e^{-b_2/x} \times \frac{1}{c_1} \\
& + \Phi(\zeta_4) \times \frac{1}{\sqrt{2\pi e^{\zeta_3}}} \exp\left(-\frac{\mu^2}{2e^{\zeta_3}}\right) \times \frac{b_2^{b_1}}{\Gamma(b_1)} e^{\zeta_3(-b_1-1)} e^{-b_2/x} \times \frac{1}{c_1} \Bigg\} \left|\det J_{T^{-1}}(\zeta)\right| \\
= \prod_{i=1}^{n} \mu_i^{y_i}(1-\mu_i)^{1-y_i} \Bigg\{ & (1-\Phi(\zeta_4)) \times \frac{1}{\sqrt{2\pi v_0 e^{\zeta_3}}} \exp\left(-\frac{\beta^2}{2v_0 e^{\zeta_3}}\right) \\
& \times \frac{b_2^{b_1}}{\Gamma(b_1)} e^{\zeta_3(-b_1-1)} e^{-b_2/x} \times \frac{1}{c_1} \\
& + \Phi(\zeta_4) \times \frac{1}{\sqrt{2\pi e^{\zeta_3}}} \exp\left(-\frac{\mu^2}{2e^{\zeta_3}}\right) \times \frac{b_2^{b_1}}{\Gamma(b_1)} e^{\zeta_3(-b_1-1)} e^{-b_2/x} \times \frac{1}{c_1} \Bigg\} \\
& \times \frac{c_1}{4\pi\sqrt{e^{a^3}}} \exp\left(-\frac{\zeta_1^2+\zeta_4^2}{2} + \zeta^3\right).
\end{aligned} \tag{5}
$$

where $(\zeta_1, \zeta_2, \zeta_3, \zeta_4) = (\Phi^{-1}(\frac{\eta}{c_1}), \beta, log(\tau^2), \Phi^{-1}(w))$, and $J_{T^{-1}}(\zeta) = \mathbf{diag}(\frac{c_1}{\sqrt{2\pi}} e^{-\frac{a_1^2}{2}}, 1, \frac{e^{a^3}}{2\sqrt{e^{a^3}}}, \frac{1}{\sqrt{2\pi}} e^{-\frac{a_4^2}{2}})$ is the Jacobian matrix of the inverse of $T(\theta)$.

A1.2.2 Full-rank Gaussian Variational Approximation

The computation of $f(\zeta, y|x)$ can be challenging due to its complex forms, we consider a full-rank Gaussian variational approximation

$$
q(\zeta; \phi) = \text{Normal}(\zeta | \mu_q, \Sigma_q) \tag{6}
$$

for $f(\zeta, y|x)$ in (A.1) where $\phi = (\mu_q, \Sigma_q)$. In order to ensure the Σ_q to be positive semidefinite, we reparameterize the covariance matrix using a Cholesky factorization, $\Sigma_q = LL^\intercal$. Then the estimation of parameters μ_q and Σ_q from (6) can be obtained by maximizing the evidence lower bound (ELBO), which is given by

$$
\mathcal{L} = \text{E}_{\zeta \sim q(\zeta)} \left\{ \text{log} f(y, T^{-1}(\zeta)|x) + log|\text{det} J_{T^{-1}}(\zeta)| \right\} + \text{H}\left\{q(\zeta; \phi)\right\}, \tag{7}
$$

where the expectation is taken with respect to ζ, and $\text{H}\{q(\zeta; \phi)\} = -\int q(\zeta; \phi) \text{log}\{q(\zeta; \phi)\} d\zeta$.

A1.2.3 Stochastic optimization

In practice, to further improve the computational efficiency, the optimization of (7) is facilitated by stochastic optimization. Specifically, We first perform an elliptical standardization transformation, $\xi = L^{-1}(\zeta - \mu_q)$ to convert the Gaussian variational approximation into a standard Gaussian. This transformation results in a variational density free of parameters,

$$q_0(\xi) = \text{Normal}(\xi|0, \mathbb{I}),$$

where $\mathbb{I}$ is the identity matrix, and then the first term on the right hand of (7) becomes

$$\mathcal{L}(\xi) = \text{E}_{q\sim q_0}[\log\, f(Y, T^{-1}(\mu_q + L\xi)) + \log|\text{det} J_{T^{-1}}(\mu_q + L\xi)|].$$

Here, the expectation is taken with respect to a standard Gaussian probability density function. Then, stochastic gradient ascent is employed to maximize (7), and we will have,

$$\frac{\partial}{\partial \mu_q}\mathcal{L} = \text{E}\left[\frac{\partial}{\partial \zeta}\text{log}f(Y, T^{-1}(\zeta))\frac{\partial}{\partial \zeta}T^{-1}(\zeta) + \frac{\partial}{\partial \zeta}\text{log}|\text{det}J_{T^{-1}}(\zeta)|\right], \tag{8}$$

$$\begin{aligned}\frac{\partial}{\partial L}\mathcal{L} =& \text{E}\left[\left\{\frac{\partial}{\partial \zeta}\text{log}f(Y, T^{-1}(\zeta))\frac{\partial}{\partial \zeta}T^{-1}(\zeta) + \frac{\partial}{\partial \zeta}\text{log}|\text{det}J_{T^{-1}}(\zeta)|\right\}\xi^{\intercal}\right] \\ &+ (L^{-1})^{\intercal}.\end{aligned} \tag{9}$$

The gradients inside the expectation can be calculated with automatic numerical differentiation. The intractable expectation is computed by Monte Carlo integration. That is, we draw samples from standard multivariate Gaussian distribution and evaluate the empirical mean of the gradients within the expectation. The detailed procedure is given in Algorithm 1. In practice, we can implement ADVI using **Rstan** based on the pseudo-code given in Algorithm 1.

A2 Simulation Studies

We conduct simulation studies to assess the performance of the proposed model in parameter estimation. We compare the autologistic model (2) with the naive approach, where the

Algorithm 1 Automatic Differentiation Variational Inference (ADVI)

Set iteration number to $i = 1$

Initialize $\mu^1 = 0$

Initialize $\mathbf{L^1} = \mathrm{I}$

while ELBO above threshold **do**

Draw ξ from the Standard Multivariate Normal Distribution

Evaluate $\frac{\partial}{\partial \mu_q}\mathcal{L}$ using equation (8)

Evaluate $\frac{\partial}{\partial L}\mathcal{L}$ using equation (9)

state stepsize s^i (a pre-specified value)

$\mu^{i+1} \leftarrow \mu^i + \mathrm{diag}(s^i)\frac{\partial}{\partial \mu_q}\mathcal{L}$

$L^{i+1} \leftarrow L^i + \mathrm{diag}(s^i)\frac{\partial}{\partial L}\mathcal{L}$

Increment iteration number

end while

Return $\hat{\mu} \leftarrow \mu^i$

Return $\hat{L} \leftarrow L^i$

spatial patterns are disregarded. We examine three distinct scenarios: in Scenario 1, we consider all the spots are displayed on a single slice; in Scenario 2, we consider that a more complex data structure with the spots may come from different slices; and the third scenario includes the situation where missing data present.

For each estimator, we report the average bias (denoted "avgBias"), the average empirical standard error (denoted "avgSEE"), the average model standard error (denoted "avgSEM"), the average coverage rate (denoted "avgCR") for 95 percent posterior credible intervals of the model parameters among 200 simulation iterations.

A2.1 Simulation 1: a Single Slice Scenario

In this simulation study, we consider the sample spots are displayed on a 30×30 lattice and for each spot (indexed as i), we generate 20 continuous covariates X_{ij}, independently from a standard normal distribution, for $j = 1, ..., 20$. A Gibbs sampler is used to simulate Y_i with the parameters $\beta = (1, 2, 3, -4, -5, \widetilde{0}_{15})^\intercal$, where $\widetilde{0}_{15}$ is a 15-dimensional zero vector. For each simulation, a random starting lattice is generated, followed by the execution of a total of 2000 iterations of the Gibbs sampler. The last iteration was taken as the realization of the lattice. Consistent with Hughes et al. (2011), we evaluate model (2) with varying autocorrelation factors $\eta = 0.4$, 1.6, and 2.8, representing low, medium, and high autocorrelation, respectively (Sun & Clayton, 2008). We set c_1 in (6) to be 8. Exemplary simulated outcomes on the lattices are displayed in Figures B1. Furthermore, we take $b_1 = 5, b_2 = 50$, and $v_0 = 0.000001$, a small value close to 0, to provide a weakly informative prior for τ, which is consistent with Ishwaran & Rao (2005).

We evaluate the performance of the proposed autologistic model (2), implemented using both NUTS and ADVI, in comparison to the naive model, which assumes $\eta_{ij} = 0$ in (1) for all i and j, meaning that disease status on the tissue is not spatially correlated. The results for bias are presented in Figures B3 and B4, the detailed results in "avgBias", "avgSEE", "avgSEM" and "avgCR" are shown in Tables C1-C3. It can be seen that the naive model generally produces a larger bias in the parameters. On the contrary, the proposed model adjusts for the spatial effects, consistently resulting in a reduced average bias for parameter

estimations regardless of the degree of the spatial correlation.

It was noteworthy that the biases of the sampling method are generally lower than that of variational inference in this context. Both methods demonstrate reasonable performance in estimating standard error in various settings, maintaining a valid coverage rate. However, the variational inference method demonstrates a much higher computational efficiency as reflected by the computing time. As shown in Figure B2, while NUTS necessitates 23 hours to execute a single simulation study, ADVI requires mere 5 minutes. Consequently, in the following simulations and data analysis on a larger scale data set, ADVI will be exclusively employed for implementation.

To assess the impact of choosing different neighbourhood radii δ in the analysis, we carry out a sensitivity analysis. Maintaining the same data-generating setup, we increase the true value of δ from 1 to 2, which corresponds to twelve neighbours per spot. To ensure comparability across settings, we scale the interaction strengths to $\eta \in \{1.2, 4.8, 8.4\}$, matching the effective signal levels under $\delta = 1$ with $\eta \in \{0.4, 1.6, 2.8\}$. The resulting biases of the estimated parameters are shown in Figure B5. We find that the parameter estimates under $\delta = 1$ and $\delta = 2$ yield similar levels of bias. In contrast, setting $\delta = 3$, which includes 28 spots in the neighbor set, leads to noticeably larger biases. These results are consistent with earlier work (F. Dormann et al., 2007), which suggests that autologistic models are most effective when restricted to relatively smaller neighbourhoods, typically involving only the first or second-order neighbours. Taken together, these findings support the use of $\delta = 1$ as a robust and conservative default across a variety of settings. [R1-C2]

To evaluate the robustness of our model under non-isotropic conditions, we conduct a simulation study in which the data are generated with differing horizontal and vertical effects across neighboring units. Specifically, taking $\delta = 1$, we simulate data by modifying $\frac{\eta}{|\mathcal{N}(i)|} \sum_{j \in \mathcal{N}(i)} Y_j$ in (2) such that η is set to be 0.4 for j in the horizontal adjacent spots and 0.7 for j in the vertical adjacent spots. The resulting biases in the parameter estimates are presented in Figure B6. Notably, the estimation performance under this anisotropic setting remained comparable to that observed in the isotropic scenario, with no substantial decrease in accuracy. [R2-C5-A3]

A2.2 Simulation 2: Evaluation of Model with Multiple Slices

In this simulation study, we assess the effectiveness of our proposed model in simultaneously handling multiple slices by incorporating the correlation between slices from the same participant. For each slice, we generate X_{gij} independently in the same way as in Section A2.1, and we consider the number of slices $G = 6$. Same as in Section A2.1, a Gibbs sampler is employed to generate Y_{gi} for $g = 1, \ldots, 6$, and $i = 1, \ldots, 900$, with $\eta = 1.6$, β to be the same as in Section A2.1, and U_{cg} are generated from $N(0, \Sigma_c)$ where $\{\sigma^2, \rho\}$ are chosen to be $\{0.1, 0.1\}$, $\{0.1, 0.4\}$, $\{0.4, 0.1\}$, $\{0.1, 0.4\}$, respectively, which reflects the different levels of variance and correlation among slices. A total of 200 simulation studies were conducted by ADVI, and we consider the same values of b_1, b_2, and v_0 as in Section A2.1.

The results for bias are reported in Figure B7 and Table C4. It can be seen that our model performs reasonably in both point estimation and variation estimation regardless of different levels of variations and correlations among slices.

In practice, the sequential ordering of tissue slices is often unavailable in publicly shared datasets. As a result, an exchangeable correlation structure is commonly assumed to account for between-slice dependencies. To assess the potential impact of misspecifying this structure, we conduct a sensitivity analysis comparing the performance of models assuming exchangeable when autoregressive ($AR(1)$) correlation structure is the truth. We repeat the simulation study under the same data-generating mechanism, except that the true correlation structure is set to AR(1). We then compare the performance of models fitted under both exchangeable and AR(1) assumptions. The results are presented in Figure B8. We observe no substantial differences in bias between the two methods, indicating that point estimation remains stable under misspecification. However, the empirical standard errors were notably higher when the correlation structure was incorrectly specified. These findings are consistent with previous theoretical results (Wakefield et al., 2013), which suggest that model misspecification in the correlation structure primarily affects efficiency rather than introducing bias. Accordingly, while the exchangeable structure remains a reasonable default in the absence of sequential information, its use may result in some loss of efficiency. [R2-C7]

A2.3 Simulation 3: Evaluation of Model with Missing Data

In this subsection, we evaluate the performance of the proposed method, where missing data are presented.

The covariates and true responses are generated in the same way as in Section A2.1. We examine two simulation scenarios for introducing missing in data under the assumption of ignorable and nonignorable missing data mechanisms, respectively. To introduce in-sample missingness in the ignorable setting, we randomly mask spots within the lattice that are not on the periphery, setting their corresponding R_i values to be 0. We considered the number of masked spots to be 10 and 30 respectively, reflecting varying degrees of missingness. For nonignorable missing mechanisms, Gibbs sampler is employed to generate R_i according to model (5) with $(\gamma_0, \gamma_1, \gamma_2)^\intercal = (-6, 1, 4)^\intercal$ and $(\gamma_0, \gamma_1, \gamma_2)^\intercal = (-5, 1, 1.6)^\intercal$, which on average will result in 1.1% and 3.7% of the total 900 spots being missing, respectively, making the number of missing spots in the nonignorable setting comparable to that in the ignorable setting. A total of 200 simulation studies are conducted.

The results for ignorable setting is reported in Figure B9, Table C5 and those for nonignorable setting are shown in Figure B10 and Table C6. Comparing with the method outlined in Section Main Model, we observe that when the number of missing points is low (e.g., 10) in the ignorable missing setting, the method exhibits a similar capability to the one described in Section Main Model. It is seen that the bias of the model may be relevant to the number of missing spots as reflected when the number of missing points reaches 30. Nonetheless, both models demonstrate comparable performance to model (2) maintaining a valid coverage rate, and are far better estimation bias than the naive model. For the nonignorable missing mechanism, we observe similar patterns as found in the ignorable setting, demonstrating the performance of the proposed model in handling the complex missing mechanism.

A3 Analysis of Alzheimer's disease (AD) mouse brain STARmap data

We analyze an imaging-based STARmap PLUS dataset from mouse brain tissue with Alzheimer's disease pathology (Zeng et al., 2023). The dataset includes AD-model mice at 8 and 13 months of age, each with two biological replicates, as well as matched controls. Following in line with the findings from Zeng et al. (2023); Lee et al. (2021), neuronal damage assessed through disintegrative staining or volumetric MRI becomes detectable around the age of nine months. Accordingly, we focused our analysis on the two replicas of the 13-month disease samples. The analysis primarily focuses on amyloid-β (Aβ) as the disease marker of AD progression in the cortical and hippocampal regions. The cells located within 64 pixels (approximately 20 μm) of a plaque center are designated as disease-associated. For neighborhood definition, we use a 60-pixel radius, which yields, on average, approximately four neighbors per spot in this subcellular-resolution dataset.

We first analyze Replicate 1 of the 13-month-old disease mouse dataset, following the same preprocessing steps described in Zeng et al. (2023). Using DiSTect, we identify eight genes that exhibit statistically significant associations with disease progression, as illustrated in Figure B12 (a). Notably, the top four genes selected by our method, including *Cst7* ($\hat{\beta} = 1.580$, 95% CrI = [1.551, 1.609]), *Trem2* ($\hat{\beta} = 0.992$, 95% CrI = [0.957, 1.027]), *C1qa* ($\hat{\beta} = 0.726$, 95% CrI = [0.693, 0.759]), and *Gfap* ($\hat{\beta} = 0.577$, 95% CrI = [0.527, 0.627]), are also identified as potential disease-associated genes in Zeng et al. (2023). In Figure B12 (b), we further investigate gene-gene interaction effects involved in disease development. From a statistical standpoint, we observe that the expression level of Hexb ($\hat{\beta} = 0.022$, 95% CrI = [0.011, 0.033]) modulates the influence of Trem2 on disease progression.

Furthermore, we assess the predictive performance of DiSTect in comparison to other competing methods, including random forest, Giotto, and MERINGUE. Consistent with the analysis conducted on HER2-positive breast cancer, we utilize the Gini-based method implemented in Giotto (Dries et al., 2021) to select eight differentially expressed genes (DEGs), matching the number of genes selected by DiSTect, and employ MERINGUE (Miller et al., 2021) to identify eight spatially variable genes (SVGs). On our 24 GB RAM system, the memory requirements for SpatialDE, PROST, and Celina at subcellular resolution exceeded available resources, so these methods were not included in this evaluation. For DiSTect, Giotto, and MERINGUE, we construct two-way interaction models using the corresponding

selected genes respectively. The training dataset consists of Replicate 1 of the 13-month disease mouse, while Replicate 2 serves as the validation dataset. We present the ground truth and predicted labels in Figure B12 (c-g), where, consistent with the HER2-positive breast cancer analysis, a cutoff of 0.5 on the estimated probability $\hat{\mu}$ is used to obtain deterministic predictions. DiSTect achieves the highest classification accuracy of 97.1%, closely followed by Giotto with an accuracy of 96.8%. However, we observe that the model based on genes selected by MERINGUE fails to produce any positive predictions, that is, no spot has $\hat{\mu} > 0.5$, suggesting that the SVGs identified by MERINGUE are not associated with disease progression in this dataset. [R2-C10-A2]

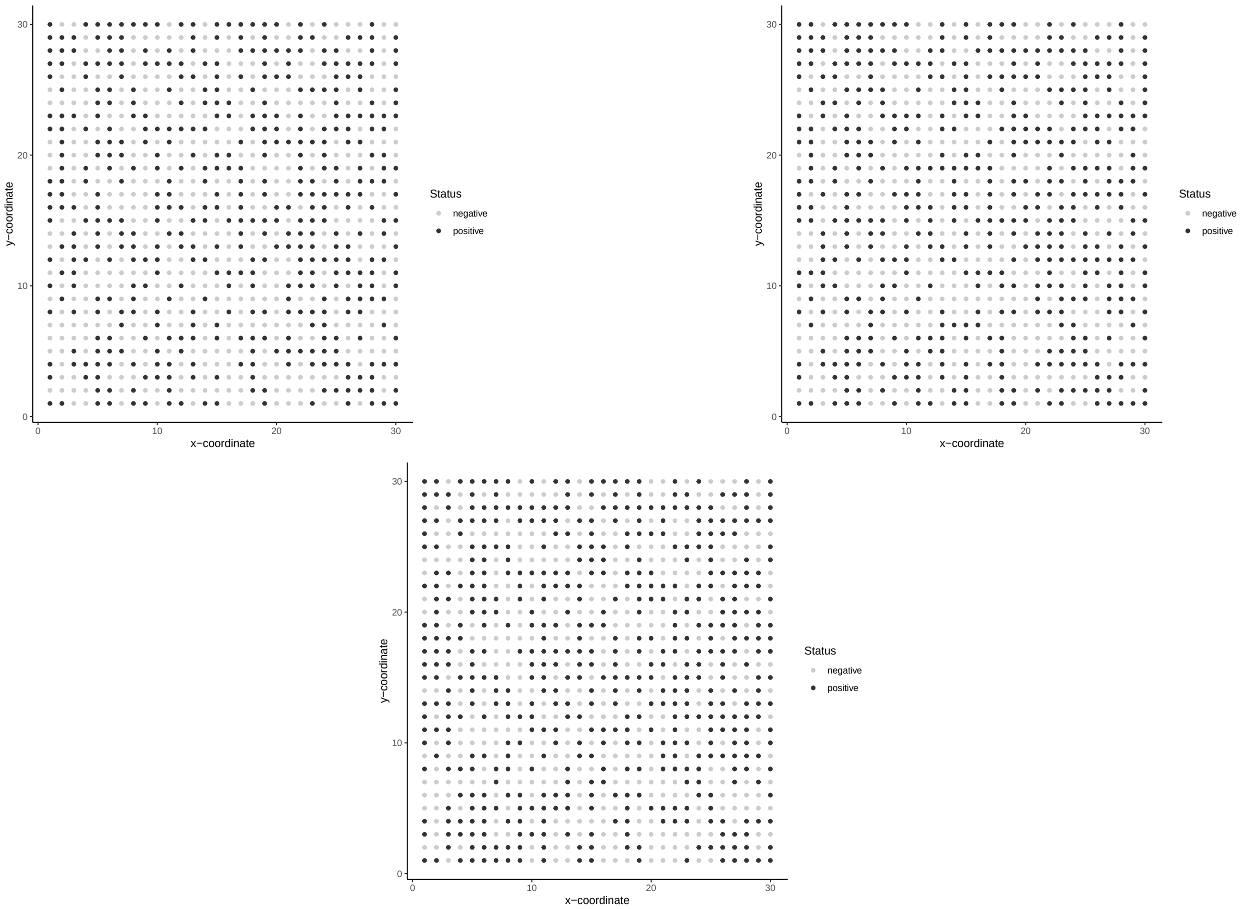

Figure B1: Exemplary figures of the generated disease outcomes in a 30×30 lattice in Simulation 1, under the scenarios of $\eta = 0.4, 1.6, 2.8$, respectively.

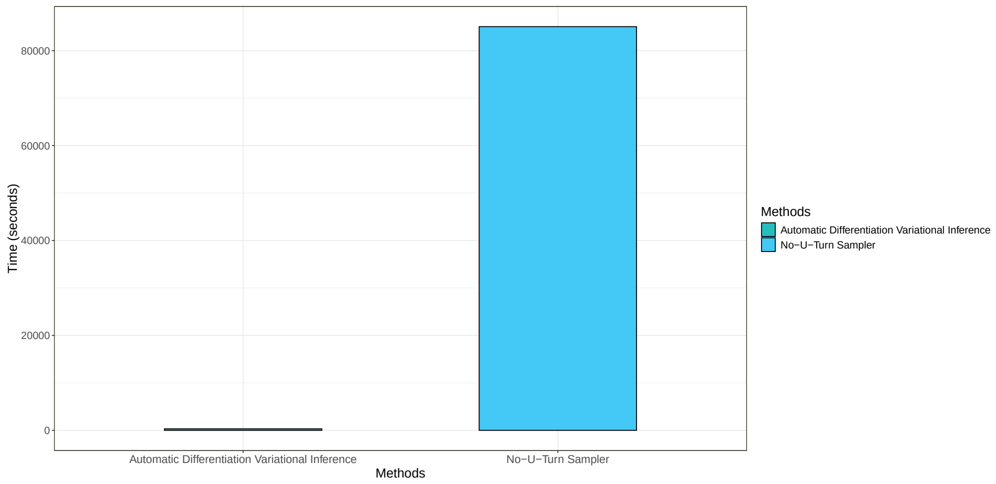

Figure B2: Comparison of running time (in seconds) for the parameter estimation of (2) implemented by ADVI and NUTS, respectively.

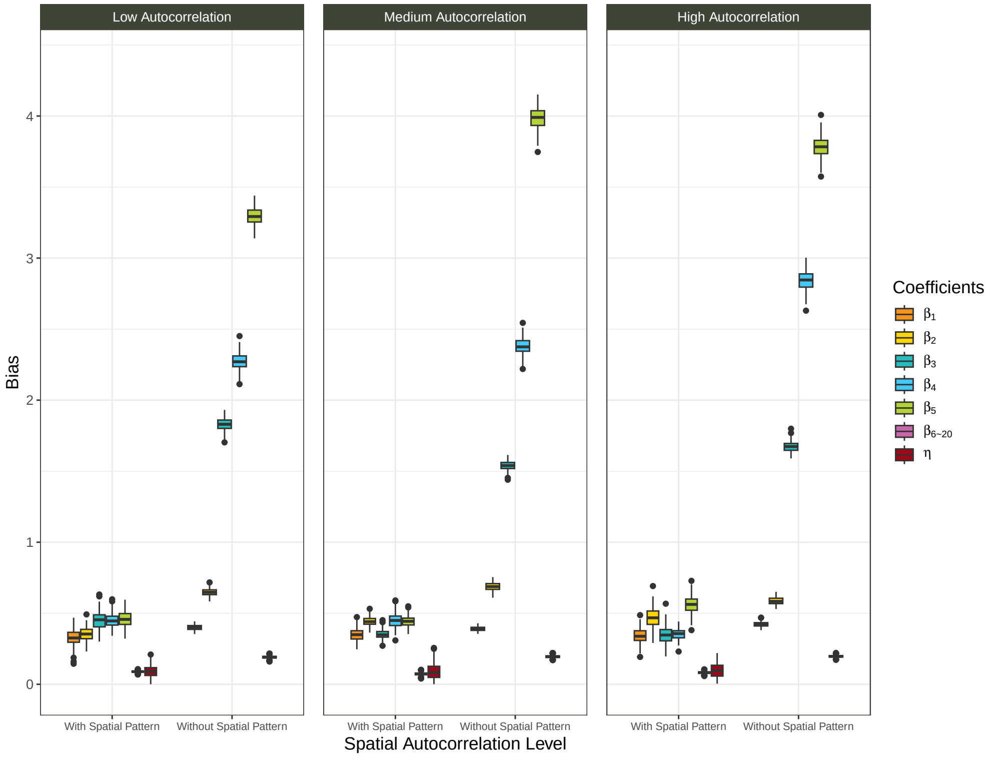

Figure B3: Boxplot of the biases for the parameter estimation in Simulation 1 implemented by ADVI algorithm. The parameters β_1 to β_5 have non-zero true values, while β_{6-20} represent the remaining parameters with true values of zero, η indicates the parameter of spatial correlation, and their boxes show their distributions of bias.

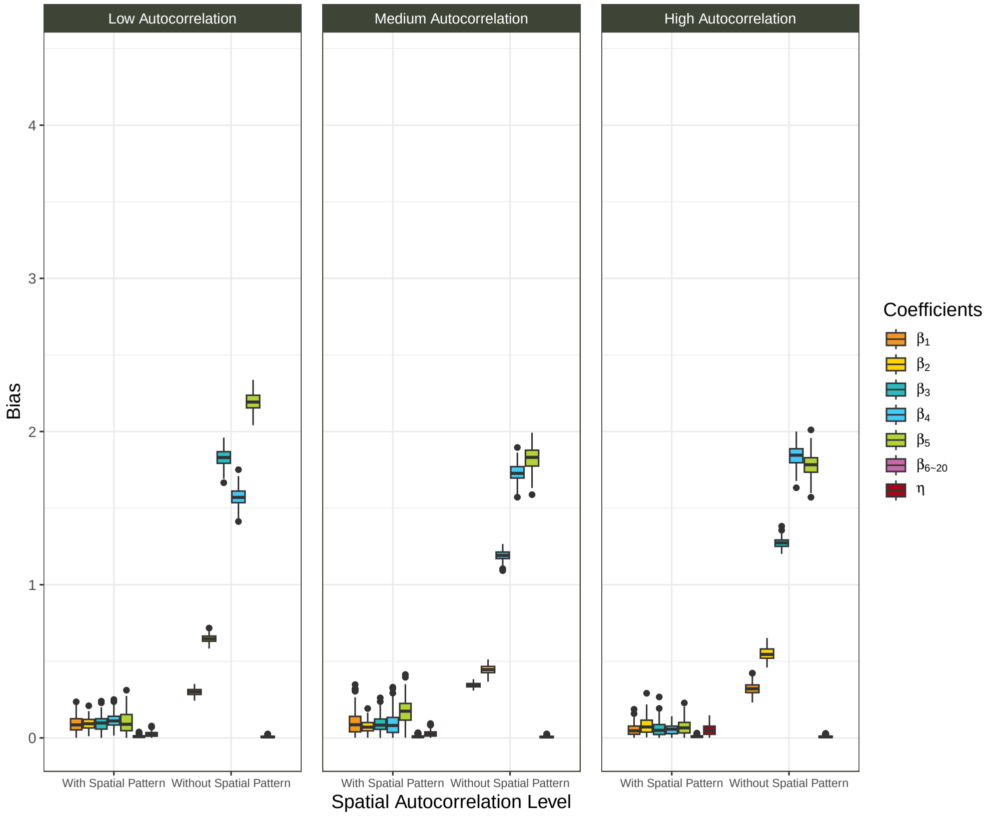

Figure B4: Boxplot of the biases for the parameter estimation in Simulation 1 implemented by NUTS algorithm. The parameters β_1 to β_5 have non-zero true values, while β_{6-20} represent the remaining parameters with true values of zero, η indicates the parameter of spatial correlation, and their boxes show their distributions of bias.

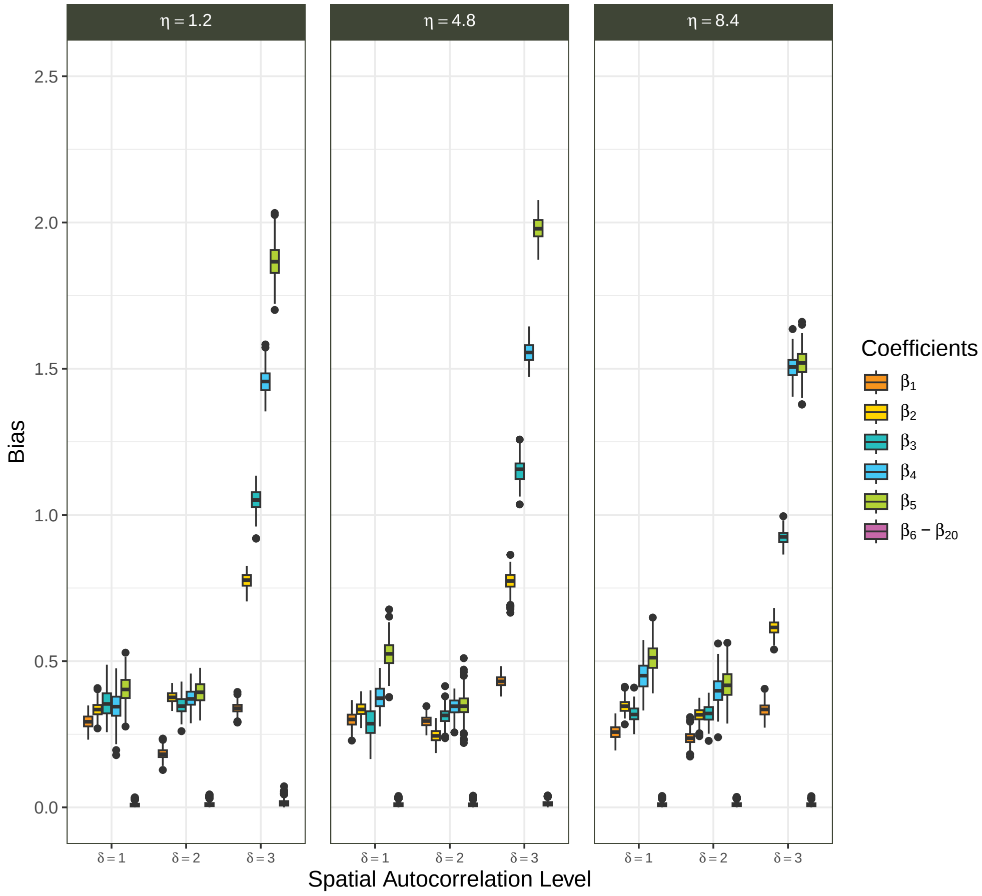

Figure B5: Boxplot of the biases for the parameter estimation in Simulation 1, illustrating the sensitivity of parameter estimates to the choice of the neighbourhood size δ. The parameters β_1 to β_5 have non-zero true values, while β_{6-20} represent the remaining parameters with true values of zero.

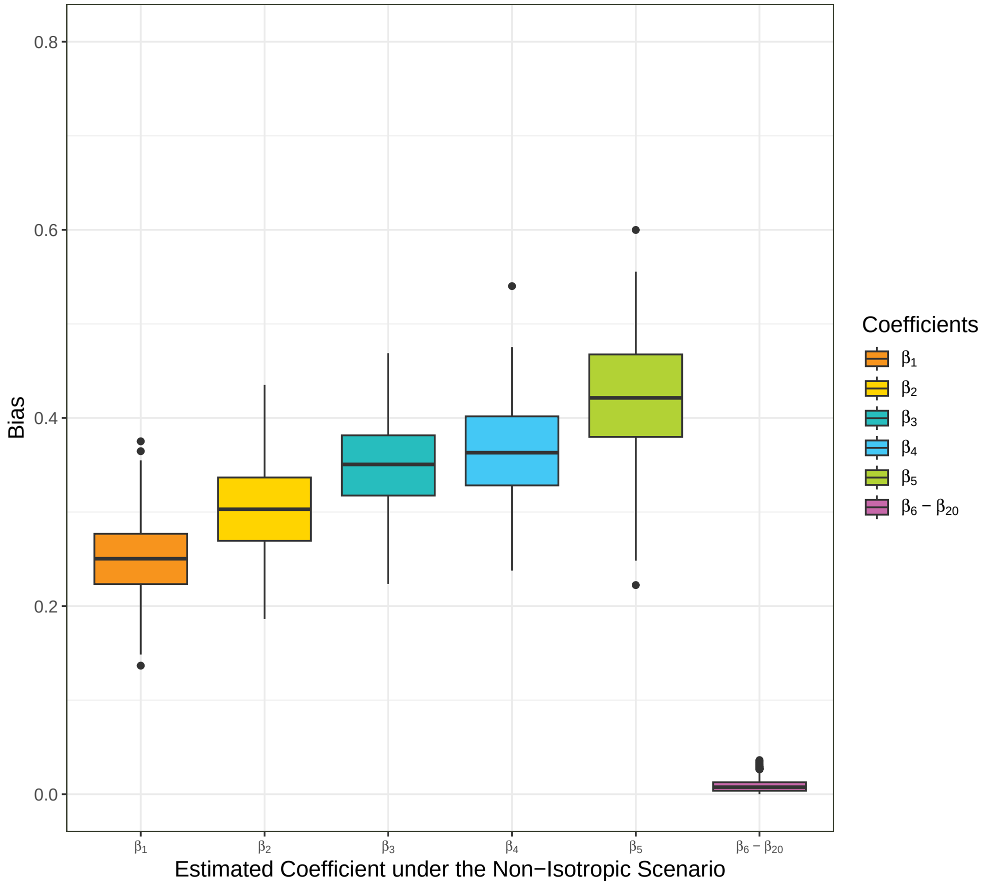

Figure B6: Boxplot of the biases for the parameter estimation in Simulation 1, illustrating the sensitivity of parameter estimates to the isotropic assumption. The parameters β_1 to β_5 have non-zero true values, while β_{6-20} represent the remaining parameters with true values of zero.

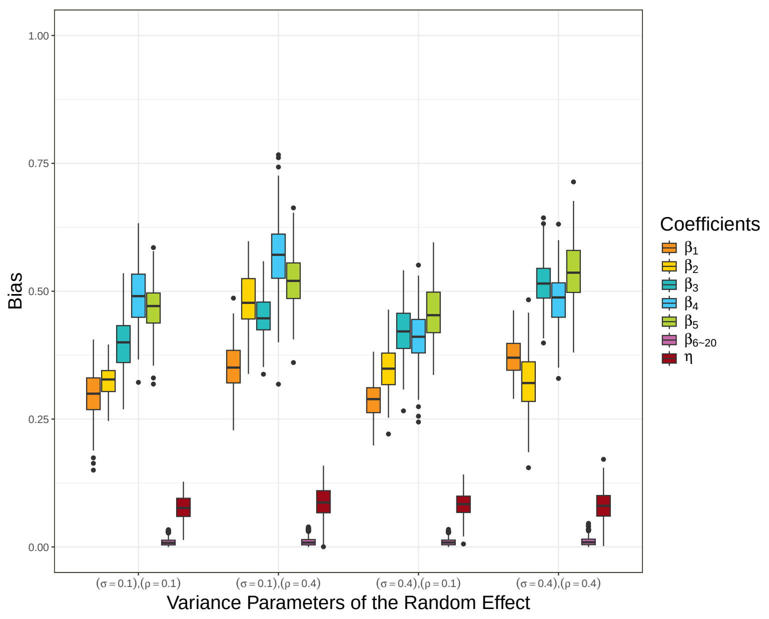

Figure B7: Boxplot of the biases for parameter estimation in Simulation 2 with varying choices of ρ and σ. The parameters β_1 to β_5 have non-zero true values, while β_{6-20} represent the remaining parameters with true values of zero, η indicates the parameter of spatial correlation, and their boxes show their distributions of bias.

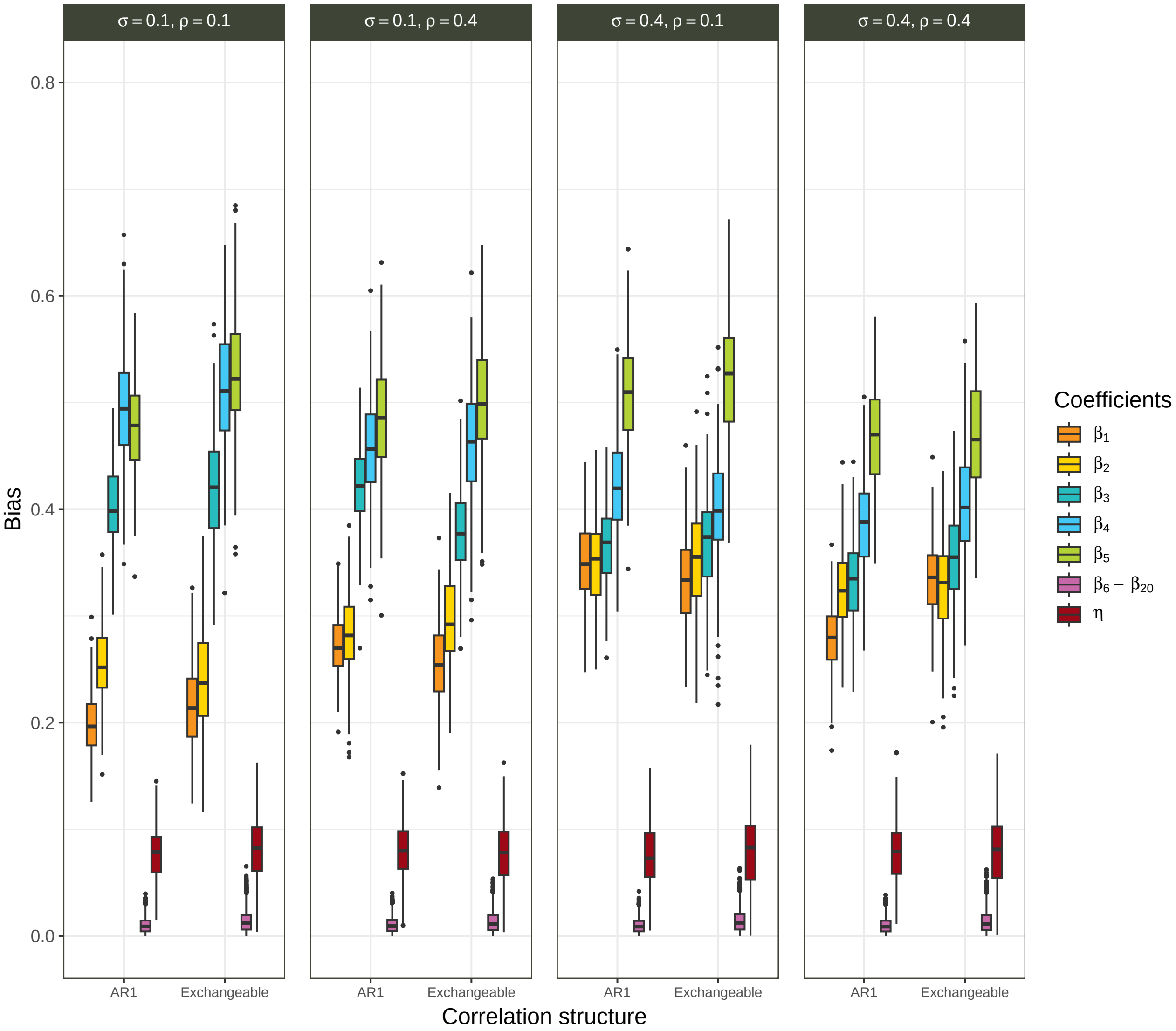

Figure B8: Boxplot of the biases for parameter estimation in Simulation 2 with varying choices of ρ and σ, illustrating the sensitivity of the model to the assumed correlation structure. The parameters β_1 to β_5 have non-zero true values, while β_{6-20} represent the remaining parameters with true values of zero, η indicates the parameter of spatial correlation, and their boxes show their distributions of bias.

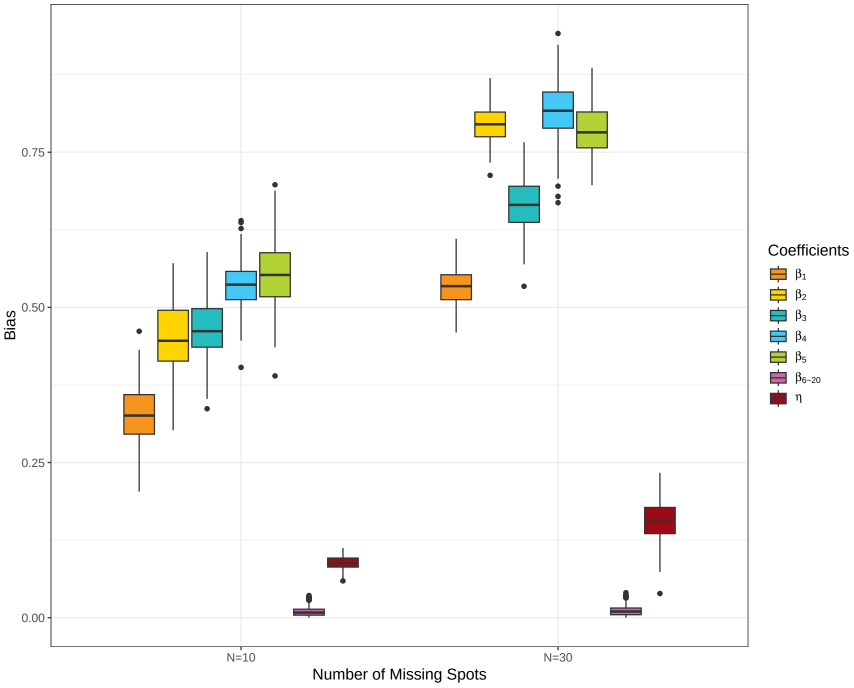

Figure B9: Boxplot of the biases for parameter estimation in Simulation 3 with ignorable missing mechanism. The parameters β_1 to β_5 have non-zero true values, while β_{6-20} represent the remaining parameters with true values of zero, η indicates the parameter of spatial correlation, and their boxes show their distributions of bias.

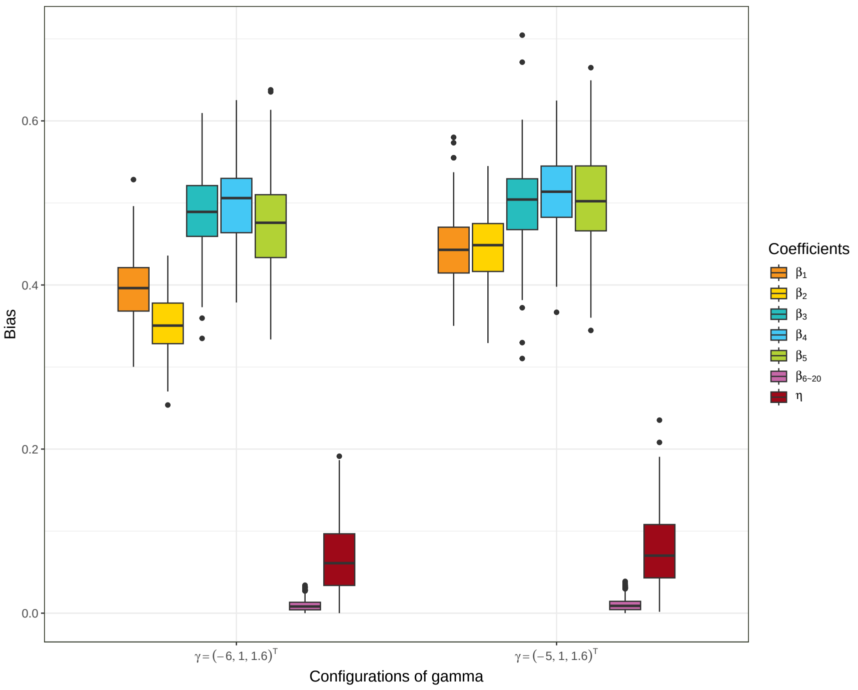

Figure B10: Boxplot of the biases for parameter estimation in Simulation 3 with nonignorable missing mechanism. The parameters β_1 to β_5 have non-zero true values, while β_{6-20} represent the remaining parameters with true values of zero, η indicates the parameter of spatial correlation, and their boxes show their distributions of bias.

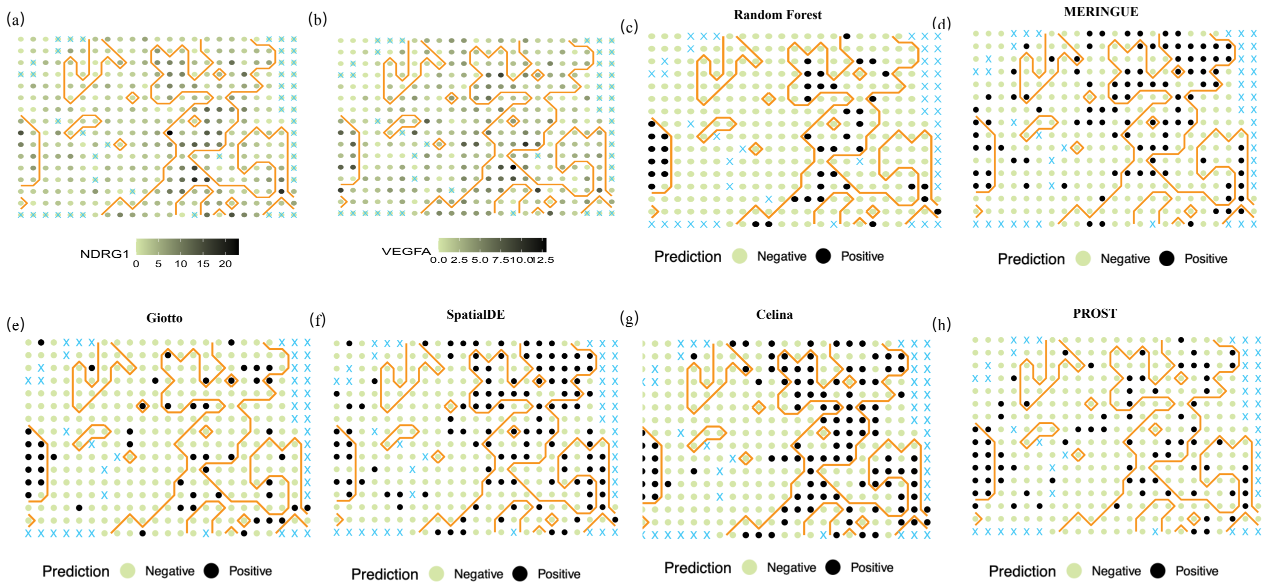

Figure B11: Exemplary spatial expression and disease predictions across methods. (a,b) Spatial expression of *NDRG1* and *VEGFA*; darker dots indicate higher normalized expression at each spot. The orange contour delineates the ground-truth boundary between cancerous and non-cancerous regions; blue x marks missing spots. (c–h) Thresholded disease predictions (probability > 0.5) from Random Forest (c), MERINGUE (d), Giotto (e), SpatialDE (f), Celina (g), and PROST (h). Black dots denote spots predicted as cancer; green dots denote non-cancer. The orange contour and blue x symbols are as in panels (a,b).

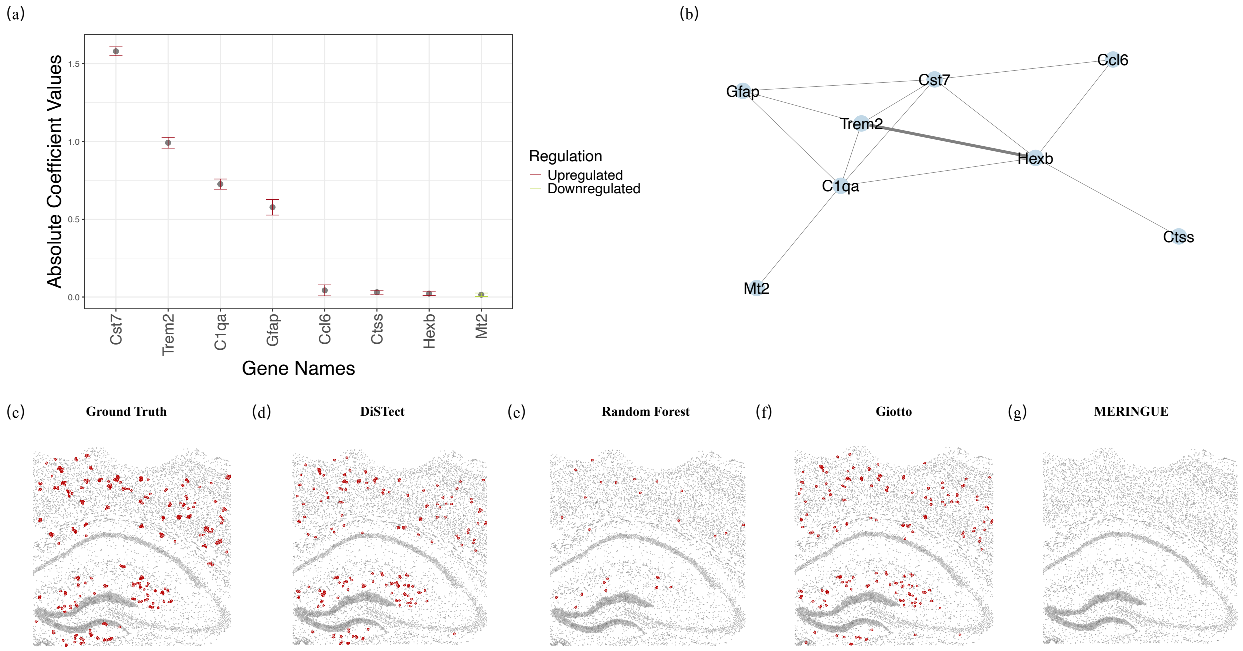

Figure B12: STARmap PLUS Alzheimer's analysis: coefficients, interactions, and predictions. (a) Posterior absolute coefficient values ($|\hat{\beta}|$) with 95% credible intervals for the eight top-ranked genes; color indicates direction of association (red, upregulated; green, downregulated). (b) Pairwise interaction network among these genes; an edge is drawn when the standardized interaction effect (posterior mean/posterior s.d.) exceeds 1.96, with edge width proportional to effect magnitude. (c) Ground-truth distribution of Aβ plaques in cortex and hippocampus for Replicate 2. (d–g) Predicted plaque-associated cells from DiSTect (d), Random Forest (e), Giotto DEGs (f), and MERINGUE SVGs (g). Red marks denote cells predicted as disease (probability > 0.5); background grayscale shows tissue context. All predictions are evaluated on Replicate 2 using models fit on Replicate 1.

Table C1: Results of Simulation for naive method and proposed methods implemented by NUTS and ADVI respectively, with lower correlation $\eta = 0.4$

	Naive Method (NUTS)				Proposed Method (NUTS)				Naive Method (ADVI)				Proposed Method(ADVI)			
	avgBias	avgSEE	avgSEM	avgCI	avgBias	avgSEE	avgSEM	avgCI	avgBias	avgSEE	avgSEM	avgCI	avgBias	avgSEE	avgSEM	avgCI
β_1	0.301	0.023	0.024	94.5%	0.089	0.016	0.016	96.0%	0.401	0.019	0.018	95.0%	0.332	0.055	0.056	95.5%
β_2	0.648	0.024	0.025	95.0%	0.092	0.021	0.022	96.0%	0.648	0.025	0.025	95.5%	0.353	0.047	0.047	95.5%
β_3	1.830	0.049	0.047	94.5%	0.095	0.027	0.028	95.5%	1.830	0.038	0.038	94.5%	0.451	0.065	0.066	95.0%
β_4	1.574	0.059	0.059	95.5%	0.11	0.037	0.037	95.0%	2.274	0.059	0.058	96%	0.445	0.047	0.047	95.0%
β_5	2.196	0.063	0.064	96.0%	0.088	0.041	0.043	96.5%	3.296	0.064	0.066	95.0%	0.458	0.056	0.057	94.5%
β_6	0.007	0.006	0.005	96.0%	0.006	0.005	0.004	96.0%	0.139	0.008	0.008	96.0%	0.005	0.005	0.004	96.5%
β_7	0.008	0.006	0.007	95.5%	0.007	0.005	0.005	97.5%	0.184	0.007	0.006	96.0%	0.006	0.005	0.008	94.0%
β_8	0.005	0.007	0.007	96.5%	0.006	0.005	0.005	96.0%	0.154	0.005	0.006	97.0%	0.004	0.005	0.004	95.5%
β_9	0.007	0.005	0.007	97.0%	0.006	0.006	0.005	93.0%	0.293	0.007	0.005	95.0%	0.006	0.006	0.005	95.5%
β_{10}	0.005	0.006	0.006	95.0%	0.005	0.005	0.006	95.5%	0.231	0.006	0.005	94.0%	0.005	0.005	0.004	95.0%
β_{11}	0.006	0.008	0.010	94.5%	0.006	0.005	0.006	96.0%	0.142	0.006	0.06	95.5%	0.006	0.004	0.004	96.0%
β_{12}	0.004	0.007	0.008	97.0%	0.006	0.004	0.005	95.0%	0.185	0.008	0.007	95.0%	0.005	0.005	0.006	95.0%
β_{13}	0.005	0.005	0.006	95.0%	0.004	0.005	0.006	95.5%	0.283	0.008	0.008	98.0%	0.004	0.006	0.006	94.0%
β_{14}	0.005	0.007	0.008	95.5%	0.007	0.006	0.007	97.0%	0.231	0.008	0.008	95.0%	0.005	0.006	0.006	93.0%
β_{15}	0.006	0.008	0.006	97.0%	0.007	0.005	0.005	95.0%	0.125	0.008	0.008	95.5%	0.005	0.004	0.005	94.5%
β_{16}	0.006	0.005	0.007	96.5%	0.006	0.006	0.006	95.0%	0.193	0.006	0.006	96.0%	0.005	0.006	0.009	96.5%
β_{17}	0.004	0.006	0.006	96.0%	0.007	0.005	0.007	94.0%	0.205	0.007	0.008	96.0%	0.005	0.005	0.006	94.5%
β_{18}	0.005	0.007	0.009	94.5%	0.006	0.006	0.005	95.0%	0.153	0.007	0.08	96.5%	0.006	0.006	0.004	95.0%
β_{19}	0.006	0.008	0.011	95.5%	0.005	0.006	0.006	94.0%	0.139	0.006	0.008	96.5%	0.005	0.006	0.007	95.0%
β_{20}	0.007	0.007	0.008	98.0%	0.006	0.006	0.007	97.0%	0.205	0.006	0.009	95.5%	0.005	0.005	0.006	94.5%
η	–	–	–	–	0.019	0.021	0.022	95.0%	–	–	–	–	0.087	0.044	0.044	95.0%

Table C2: Results of Simulation for naive method and proposed methods implemented by NUTS and ADVI respectively, with lower correlation $\eta = 1.6$

	Naive Method (NUTS)				Proposed Method (NUTS)				Naive Method (ADVI)				Proposed Method(ADVI)			
	avgBias	avgSEE	avgSEM	avgCI	avgBias	avgSEE	avgSEM	avgCI	avgBias	avgSEE	avgSEM	avgCI	avgBias	avgSEE	avgSEM	avgCI
β_1	0.343	0.016	0.018	94.0%	0.083	0.011	0.013	95.5%	0.389	0.016	0.016	95.5%	0.353	0.043	0.043	93.5%
β_2	0.447	0.023	0.027	95.0%	0.073	0.017	0.017	95.0%	0.689	0.025	0.027	95.0%	0.443	0.032	0.033	95.5%
β_3	1.192	0.034	0.034	95.0%	0.095	0.015	0.029	96.0%	1.540	0.034	0.034	94.0%	0.355	0.033	0.034	94.5%
β_4	1.738	0.067	0.065	95.5%	0.082	0.033	0.034	95.0%	2.386	0.067	0.067	95.0%	0.452	0.046	0.047	96.0%
β_5	1.823	0.069	0.069	95.0%	0.183	0.04	0.043	94.5%	3.982	0.069	0.069	95.5%	0.447	0.038	0.039	96.5%
β_6	0.005	0.004	0.004	97.0%	0.006	0.004	0.004	95.0%	0.138	0.007	0.007	95.0%	0.006	0.008	0.008	95.0%
β_7	0.008	0.006	0.007	95.0%	0.004	0.004	0.008	97.0%	0.174	0.008	0.008	94.5%	0.005	0.009	0.008	94.5%
β_8	0.005	0.009	0.009	96.5%	0.005	0.005	0.004	95.5%	0.164	0.006	0.007	96.0%	0.008	0.009	0.009	96.5%
β_9	0.006	0.007	0.009	96.5%	0.004	0.004	0.005	96.5%	0.193	0.006	0.007	97.0%	0.007	0.008	0.009	95.0%
β_{10}	0.005	0.007	0.007	93.0%	0.006	0.004	0.004	96.0%	0.271	0.006	0.006	96.0%	0.007	0.007	0.009	95.5%
β_{11}	0.005	0.007	0.011	97.5%	0.006	0.004	0.004	98.0%	0.192	0.008	0.08	95.0%	0.008	0.008	0.009	95.5%
β_{12}	0.007	0.006	0.007	96.0%	0.007	0.004	0.006	95.5%	0.185	0.006	0.007	95.5%	0.007	0.006	0.006	95.5%
β_{13}	0.007	0.008	0.007	96.0%	0.005	0.006	0.006	96.0%	0.283	0.006	0.006	96.0%	0.007	0.007	0.005	94.5%
β_{14}	0.006	0.008	0.008	96.0%	0.005	0.004	0.006	95.0%	0.131	0.007	0.007	95.5%	0.007	0.005	0.005	96.0%
β_{15}	0.007	0.007	0.008	94.5%	0.006	0.005	0.005	95.5%	0.222	0.007	0.006	95.5%	0.006	0.009	0.007	95.0%
β_{16}	0.007	0.007	0.008	94.0%	0.005	0.005	0.009	95.0%	0.187	0.007	0.007	97.0%	0.007	0.009	0.008	94.5%
β_{17}	0.008	0.006	0.008	97.0%	0.005	0.004	0.006	94.0%	0.195	0.006	0.006	94.5%	0.007	0.009	0.008	95.0%
β_{18}	0.005	0.007	0.013	94.0%	0.004	0.004	0.004	96.5%	0.183	0.008	0.008	94.0%	0.006	0.009	0.009	95.0%
β_{19}	0.006	0.007	0.008	95.5%	0.005	0.005	0.007	94.0%	0.169	0.006	0.006	95.5%	0.008	0.008	0.008	95.0%
β_{20}	0.006	0.007	0.009	98.0%	0.005	0.005	0.006	95.5%	0.198	0.008	0.007	95.0%	0.008	0.009	0.009	95.5%
η	–	–	–	–	0.021	0.028	0.030	96.0%	–	–	–	–	0.083	0.069	0.070	95.0%

Table C3: Results of Simulation for naive method and proposed methods implemented by NUTS and ADVI respectively, with lower correlation $\eta = 2.8$

	Naive Method (NUTS)				Proposed Method (NUTS)				Naive Method (ADVI)				Proposed Method(ADVI)			
	avgBias	avgSEE	avgSEM	avgCI	avgBias	avgSEE	avgSEM	avgCI	avgBias	avgSEE	avgSEM	avgCI	avgBias	avgSEE	avgSEM	avgCI
β_1	0.037	0.038	0.038	95.0%	0.034	0.056	0.057	96.5%	0.423	0.017	0.017	94.0%	0.334	0.055	0.056	94.5%
β_2	0.043	0.045	0.043	97.0%	0.067	0.062	0.063	95.0%	0.582	0.027	0.027	94.5%	0.467	0.047	0.047	96.5%
β_3	1.273	0.036	0.032	95.5%	0.044	0.058	0.058	95.0%	1.673	0.038	0.037	95.5%	0.344	0.065	0.066	95.5%
β_4	1.833	0.062	0.062	94.5%	0.056	0.037	0.037	93.0%	2.833	0.063	0.063	97.0%	0.356	0.047	0.047	94.0%
β_5	1.786	0.073	0.072	95.0%	0.056	0.058	0.059	94.5%	3.786	0.072	0.073	95.5%	0.556	0.056	0.057	94.0%
β_6	0.008	0.006	0.007	96.0%	0.008	0.006	0.005	95.5%	0.133	0.006	0.006	94.0%	0.008	0.007	0.007	96.0%
β_7	0.005	0.005	0.004	96.0%	0.005	0.005	0.006	96.0%	0.144	0.007	0.007	95.5%	0.008	0.006	0.006	94.5%
β_8	0.008	0.006	0.005	94.0%	0.008	0.007	0.007	94.0%	0.184	0.006	0.007	95.0%	0.007	0.007	0.007	95.0%
β_9	0.006	0.006	0.006	94.5%	0.0008	0.005	0.006	96.0%	0.195	0.005	0.006	95.5%	0.004	0.007	0.005	95.0%
β_{10}	0.006	0.007	0.006	95.5%	0.007	0.007	0.006	96.5%	0.168	0.007	0.006	96.0%	0.005	0.007	0.007	95.5%
β_{11}	0.007	0.005	0.008	94.0%	0.006	0.004	0.006	95.0%	0.242	0.006	0.07	95.0%	0.005	0.006	0.006	94.5%
β_{12}	0.005	0.006	0.006	97.5%	0.008	0.006	0.007	95.0%	0.172	0.005	0.008	94.5%	0.007	0.007	0.008	95.5%
β_{13}	0.006	0.006	0.007	95.5%	0.009	0.007	0.006	97.0%	0.194	0.008	0.008	95.5%	0.008	0.008	0.008	93.0%
β_{14}	0.006	0.006	0.005	95.5%	0.007	0.008	0.007	95.5%	0.287	0.009	0.009	95.0%	0.007	0.005	0.005	93.5%
β_{15}	0.005	0.005	0.006	97.5%	0.008	0.008	0.008	95.0%	0.276	0.007	0.008	93.5%	0.005	0.007	0.008	95.5%
β_{16}	0.006	0.005	0.007	94.0%	0.007	0.009	0.009	94.0%	0.217	0.008	0.008	95.0%	0.007	0.007	0.005	95.5%
β_{17}	0.006	0.005	0.006	95.0%	0.008	0.006	0.007	94.5%	0.198	0.007	0.007	94.5%	0.008	0.006	0.006	95.5%
β_{18}	0.006	0.007	0.007	95.5%	0.008	0.007	0.005	96.5%	0.249	0.007	0.006	93.5%	0.007	0.008	0.008	95.5%
β_{19}	0.004	0.006	0.009	95.0%	0.008	0.005	0.006	95.0%	0.219	0.006	0.006	96.0%	0.007	0.007	0.007	96.0%
β_{20}	0.005	0.007	0.007	96.0%	0.006	0.007	0.007	94.5%	0.226	0.007	0.008	94.5%	0.007	0.007	0.007	96.5%
η	–	–	–	–	0.045	0.047	0.045	95.5%	–	–	–	–	0.095	0.057	0.056	95.5%

Table C4: Simulation results for proposed methods implemented by ADVI under different cross-tissue correlation structures

	$\sigma = 0.1, \rho = 0.1$				$\sigma = 0.1, \rho = 0.4$				$\sigma = 0.4, \rho = 0.1$				$\sigma = 0.4, \rho = 0.4$			
	avgBias	avgSEE	avgSEM	avgCI	avgBias	avgSEE	avgSEM	avgCI	avgBias	avgSEE	avgSEM	avgCI	avgBias	avgSEE	avgSEM	avgCI
β_1	0.303	0.046	0.047	95.5%	0.362	0.045	0.043	94.0%	0.288	0.034	0.034	95.0%	0.371	0.032	0.033	94.5%
β_2	0.331	0.032	0.032	94.0%	0.481	0.054	0.054	95.0%	0.351	0.045	0.045	94.0%	0.323	0.059	0.059	95.5%
β_3	0.398	0.048	0.048	95.5%	0.452	0.042	0.043	94.5%	0.424	0.046	0.045	94.0%	0.511	0.048	0.047	93.0%
β_4	0.494	0.057	0.059	96.5%	0.568	0.076	0.074	95.5%	0.412	0.057	0.054	95.0%	0.493	0.049	0.045	95.0%
β_5	0.469	0.043	0.043	95.0%	0.524	0.054	0.054	93.5%	0.459	0.063	0.063	95.5%	0.551	0.063	0.059	94.5%
β_6	0.008	0.005	0.005	94.0%	0.005	0.009	0.008	93.0%	0.006	0.008	0.008	95.0%	0.004	0.009	0.009	94.0%
β_7	0.007	0.006	0.006	94.5%	0.008	0.009	0.009	92.5%	0.007	0.009	0.007	93.0%	0.006	0.009	0.009	92.0%
β_8	0.008	0.009	0.009	97.0%	0.007	0.008	0.008	95.5%	0.009	0.009	0.009	94.5%	0.007	0.010	0.010	95.5%
β_9	0.008	0.008	0.008	96.0%	0.007	0.009	0.009	94.0%	0.006	0.009	0.009	96.0%	0.095	0.009	0.009	94.5%
β_{10}	0.006	0.008	0.008	93.0%	0.007	0.009	0.009	96.0%	0.009	0.007	0.007	95.5%	0.008	0.011	0.011	94.0%
β_{11}	0.007	0.009	0.008	95.5%	0.008	0.009	0.009	95.0%	0.008	0.009	0.010	93.5%	0.009	0.008	0.008	92.0%
β_{12}	0.007	0.009	0.007	95.0%	0.008	0.009	0.007	96.5%	0.005	0.008	0.008	94.0%	0.009	0.008	0.008	95.5%
β_{13}	0.008	0.006	0.006	93.0%	0.009	0.009	0.009	94.5%	0.009	0.008	0.007	92.0%	0.007	0.006	0.006	93.0%
β_{14}	0.008	0.008	0.008	93.5%	0.009	0.008	0.009	93.0%	0.009	0.008	0.008	93.0%	0.007	0.006	0.006	94.5%
β_{15}	0.009	0.008	0.008	94.0%	0.011	0.010	0.011	95.5%	0.008	0.009	0.009	95.5%	0.012	0.009	0.009	96.5%
β_{16}	0.009	0.008	0.007	95.5%	0.09	0.009	0.009	93.5%	0.007	0.009	0.009	96.0%	0.007	0.009	0.009	93.0%
β_{17}	0.009	0.008	0.006	94.0%	0.009	0.009	0.009	95.0%	0.007	0.008	0.008	94.0%	0.008	0.009	0.009	95.0%
β_{18}	0.008	0.006	0.006	94.0%	0.007	0.009	0.009	95.5%	0.004	0.008	0.011	96.0%	0.009	0.009	0.011	93.5%
β_{19}	0.007	0.008	0.005	93.5%	0.008	0.009	0.009	95.0%	0.004	0.008	0.008	94.5%	0.009	0.009	0.007	96.0%
β_{20}	0.005	0.009	0.008	95.0%	0.009	0.007	0.007	95.0%	0.009	0.008	0.008	94.0%	0.012	0.009	0.007	91.5%
η	0.076	0.025	0.025	94.5%	0.089	0.031	0.031	94.0%	0.083	0.022	0.022	93.5%	0.081	0.029	0.026	95.0%

Table C5: Result for Simulation in the ignorable missing setting

	10 Missing Spots				30 Missing Spots			
	avgBias	avgSEE	avgSEM	avgCI	avgBias	avgSEE	avgSEM	avgCI
β_1	0.335	0.043	0.043	95.0%	0.535	0.028	0.028	97.0%
β_2	0.449	0.056	0.057	95.0%	0.796	0.028	0.029	95.5%
β_3	0.465	0.048	0.048	95.5%	0.664	0.038	0.038	95.0%
β_4	0.536	0.039	0.039	96.5%	0.816	0.048	0.047	96.5%
β_5	0.552	0.055	0.056	93.5%	0.787	0.047	0.047	95.5%
β_6	0.009	0.006	0.006	95.0%	0.009	0.009	0.009	94.5%
β_7	0.009	0.008	0.007	96.5%	0.009	0.009	0.009	96.0%
β_8	0.008	0.006	0.006	94.0%	0.011	0.009	0.009	94.5%
β_9	0.009	0.006	0.005	95.0%	0.009	0.007	0.009	96.0%
β_{10}	0.008	0.007	0.007	96.0%	0.009	0.008	0.008	97.0%
β_{11}	0.007	0.008	0.008	96.5%	0.008	0.009	0.009	96.0%
β_{12}	0.009	0.008	0.008	93.5%	0.009	0.007	0.007	95.0%
β_{13}	0.010	0.009	0.009	96.0%	0.012	0.008	0.007	95.0%
β_{14}	0.008	0.009	0.009	97.0%	0.009	0.006	0.006	96.0%
β_{15}	0.006	0.009	0.010	94.5%	0.009	0.008	0.009	95.0%
β_{16}	0.008	0.009	0.009	97.0%	0.007	0.007	0.007	95.0%
β_{17}	0.007	0.009	0.010	94.0%	0.008	0.008	0.008	95.5%
β_{18}	0.008	0.004	0.007	97.5%	0.009	0.009	0.009	97.5%
β_{19}	0.008	0.008	0.009	95.0%	0.008	0.006	0.007	95.5%
β_{20}	0.008	0.009	0.008	95.5%	0.009	0.007	0.008	95.5%
η	0.089	0.011	0.012	95.5%	0.156	0.029	0.029	95.0%

Table C6: Result for Simulation in the nonignorable missing setting

	$(\gamma_0, \gamma_1, \gamma_2)^\intercal = (-6, 1, 4)^\intercal$				$(\gamma_0, \gamma_1, \gamma_2)^\intercal = (-5, 1, 1.6)^\intercal$			
	avgBias	avgSEE	avgSEM	avgCI	avgBias	avgSEE	avgSEM	avgCI
β_1	0.398	0.041	0.041	94.5%	0.438	0.046	0.045	95.0%
β_2	0.349	0.037	0.037	94.0%	0.451	0.048	0.047	95.5%
β_3	0.489	0.048	0.048	94.0%	0.511	0.053	0.053	96.0%
β_4	0.501	0.050	0.051	95.5%	0.519	0.042	0.045	95.5%
β_5	0.471	0.059	0.056	95.5%	0.507	0.055	0.055	92.5%
β_6	0.009	0.009	0.009	96.5%	0.008	0.008	0.008	91.5%
β_7	0.009	0.009	0.009	97.5%	0.008	0.007	0.007	93.5%
β_8	0.008	0.008	0.008	95.5%	0.009	0.009	0.009	94.5%
β_9	0.009	0.008	0.008	95.5%	0.008	0.006	0.006	95.0%
β_{10}	0.009	0.008	0.007	93.0%	0.009	0.006	0.005	94.0%
β_{11}	0.006	0.006	0.006	94.5%	0.008	0.007	0.006	95.5%
β_{12}	0.007	0.005	0.005	97.0%	0.008	0.005	0.006	95.0%
β_{13}	0.007	0.006	0.009	95.0%	0.009	0.007	0.009	94.0%
β_{14}	0.005	0.008	0.008	95.0%	0.009	0.009	0.009	93.0%
β_{15}	0.007	0.009	0.008	95.5%	0.006	0.009	0.009	94.5%
β_{16}	0.009	0.006	0.005	92.0%	0.007	0.008	0.009	95.0%
β_{17}	0.008	0.006	0.006	94.5%	0.008	0.008	0.008	95.5%
β_{18}	0.008	0.009	0.008	95.0%	0.009	0.009	0.009	94.5%
β_{19}	0.008	0.008	0.008	95.0%	0.007	0.008	0.008	95.0%
β_{20}	0.008	0.007	0.009	93.5%	0.008	0.009	0.009	94.5%
η	0.062	0.052	0.052	95.5%	0.066	0.055	0.055	95.0%

Table C7: Specification of hyperparameters in Analysis of HER2-positive Breast Cancer Spatial Transcriptomic Data

**Parameters**	**Values**
b_1	5
b_2	50
b_3	5
b_4	50
b_5	0
b_6	10
c_1	8
v_0	0.000001

References

Andersson, A., Larsson, L., Stenbeck, L., Salmén, F., Ehinger, A., Wu, S. Z., Al-Eryani, G., Roden, D., Swarbrick, A., Borg, Å., et al. (2021). Spatial deconvolution of HER2-positive breast cancer delineates tumor-associated cell type interactions. *Nature Communications*, *12*(1), 6012.

Arisdakessian, C., Poirion, O., Yunits, B., Zhu, X., & Garmire, L. X. (2019). Deepimpute: an accurate, fast, and scalable deep neural network method to impute single-cell RNA-seq data. *Genome Biology*, *20*, 1–14.

Bauer, S., Horn, D., Robinson, P., et al. (2008). Walking the interactome for prioritization of candidate disease genes. *American Journal of Human Genetics*, *82*, 949–958.

Biancalani, T., Scalia, G., Buffoni, L., Avasthi, R., Lu, Z., Sanger, A., Tokcan, N., Vanderburg, C. R., Segerstolpe, Å., Zhang, M., et al. (2021). Deep learning and alignment of spatially resolved single-cell transcriptomes with Tangram. *Nature Methods*, *18*(11), 1352–1362.

Caragea, P. C., & Kaiser, M. S. (2009). Autologistic models with interpretable parameters. *Journal of Agricultural, Biological, and Environmental Statistics*, *14*, 281–300.

Dominguez-Villar, M., & Hafler, D. A. (2018). Regulatory T-cells in autoimmune disease. *Nature Immunology*, *19*(7), 665–673.

Dong, C., Jin, Y.-T., Hua, H.-L., Wen, Q.-F., Luo, S., Zheng, W.-X., & Guo, F.-B. (2020). Comprehensive review of the identification of essential genes using computational methods: focusing on feature implementation and assessment. *Briefings in Bioinformatics*, *21*(1), 171–181.

Dormann, C. F. (2007). Effects of incorporating spatial autocorrelation into the analysis of species distribution data. *Global ecology and biogeography*, *16*(2), 129–138.

Dries, R., Zhu, Q., Dong, R., Eng, C.-H. L., Li, H., Liu, K., Fu, Y., Zhao, T., Sarkar, A.,

Bao, F., et al. (2021). Giotto: a toolbox for integrative analysis and visualization of spatial expression data. *Genome Biology*, *22*(1), 78.

Du, J., An, Z.-J., Huang, Z.-F., Yang, Y.-C., Zhang, M.-H., & Hou, J. (2023). Novel insights from spatial transcriptome analysis in solid tumors. *International Journal of Biological Sciences*, *19*(15), 4778.

Eraslan, G., Simon, L. M., Mircea, M., Mueller, N. S., & Theis, F. J. (2019). Single-cell RNA-seq denoising using a deep count autoencoder. *Nature Communications*, *10*(1), 390.

F. Dormann, C., M. McPherson, J., B. Araújo, M., Bivand, R., Bolliger, J., Carl, G., G. Davies, R., Hirzel, A., Jetz, W., Daniel Kissling, W., et al. (2007). Methods to account for spatial autocorrelation in the analysis of species distributional data: a review. *Ecography*, *30*(5), 609–628.

Feng, C., Liu, S., Zhang, H., Guan, R., Li, D., Zhou, F., Liang, Y., & Feng, X. (2020). Dimension reduction and clustering models for single-cell RNA sequencing data: a comparative study. *International Journal of Molecular Sciences*, *21*(6), 2181.

Guala, D., & Sjölund, E. (2014). Maxlink: network-based prioritization of genes tightly linked to a disease seed set. *Bioinformatics*, *30*(18), 2689–2690.

Hao, M., Hua, K., & Zhang (2021). Somde: a scalable method for identifying spatially variable genes with self-organizing map. *Bioinformatics*, *37*(23), 4392–4398.

Hoffman, M. D., Gelman, A., et al. (2014). The No-U-Turn sampler: adaptively setting path lengths in Hamiltonian Monte Carlo. *Journal of Machine Learning Research*, *15*(1), 1593–1623.

Hu, J., Li, X., Coleman, K., Schroeder, A., Ma, N., Irwin, D. J., Lee, E. B., Shinohara, R. T., & Li, M. (2021). SpaGCN: Integrating gene expression, spatial location and histology to identify spatial domains and spatially variable genes by graph convolutional network. *Nature Methods*, *18*(11), 1342–1351.

Huang, M., Wang, J., Torre, E., Dueck, H., Shaffer, S., Bonasio, R., Murray, J. I., Raj, A., Li, M., & Zhang, N. R. (2018). SAVER: gene expression recovery for single-cell RNA sequencing. *Nature Methods*, *15*(7), 539–542.

Hughes, J., Haran, M., & Caragea, P. C. (2011). Autologistic models for binary data on a lattice. *Environmetrics*, *22*(7), 857–871.

Ishwaran, H., & Rao, J. S. (2005). Spike and slab variable selection: frequentist and bayesian strategies. *The Annals of Statistics*, *33*(2), 730–773.

Jia, P., Zheng, S., Long, J., Zheng, W., & Zhao, Z. (2011). dmGWAS: dense module searching for genome-wide association studies in protein–protein interaction networks. *Bioinformatics*, *27*(1), 95–102.

Jiang, R., Li, Z., Jia, Y., & Li, S. (2023). SINFONIA: scalable identification of spatially variable genes for deciphering spatial domains. *Cells*, *12*(4), 604.

Kucukelbir, A., Tran, D., Ranganath, R., Gelman, A., & Blei, D. M. (2017). Automatic differentiation variational inference. *Journal of Machine Learning Research*, *18*(14), 1–45.

Lauritzen, S. L. (1996). *Graphical Models*, vol. 17. Clarendon Press.

Lee, S.-H., Meilandt, W. J., Xie, L., Gandham, V. D., Ngu, H., Barck, K. H., Rezzonico, M. G., Imperio, J., Lalehzadeh, G., Huntley, M. A., et al. (2021). Trem2 restrains the enhancement of tau accumulation and neurodegeneration by β-amyloid pathology. *Neuron*, *109*(8), 1283–1301.

Leiserson, M. D., Vandin, F., Wu, H.-T., Dobson, J. R., Eldridge, J. V., Thomas, J. L., Papoutsaki, A., Kim, Y., Niu, B., McLellan, M., et al. (2015). Pan-cancer network analysis identifies combinations of rare somatic mutations across pathways and protein complexes. *Nature Genetics*, *47*(2), 106–114.

Li, X., & Wang, C.-Y. (2021). From bulk, single-cell to spatial RNA sequencing. *International Journal of Oral Science*, *13*(1), 36.

Li, Z., & Zhou, X. (2022). BASS: multi-scale and multi-sample analysis enables accurate cell type clustering and spatial domain detection in spatial transcriptomic studies. *Genome Biology*, *23*(1), 168.

Liang, Y., Shi, G., Cai, R., Yuan, Y., Xie, Z., Yu, L., Huang, Y., Shi, Q., Wang, L., Li, J., et al. (2024). Prost: quantitative identification of spatially variable genes and domain detection in spatial transcriptomics. *Nature Communications*, *15*(1), 600.

Linderman, G. C., Zhao, J., Roulis, M., Bielecki, P., Flavell, R. A., Nadler, B., & Kluger, Y. (2022). Zero-preserving imputation of single-cell RNA-seq data. *Nature Communications*, *13*(1), 192.

Lopez, R., Nazaret, A., Langevin, M., Samaran, J., Regier, J., Jordan, M. I., & Yosef, N. (2019). A joint model of unpaired data from scRNA-seq and spatial transcriptomics for imputing missing gene expression measurements. *arXiv preprint. arXiv:1905.02269*.

Lopez, R., Regier, J., Cole, M. B., Jordan, M. I., & Yosef, N. (2018). Deep generative modeling for single-cell transcriptomics. *Nature Methods*, *15*(12), 1053–1058.

Martin, J. B. (1999). Molecular basis of the neurodegenerative disorders. *New England Journal of Medicine*, *340*(25), 1970–1980.

Marx, V. (2021). Method of the Year: spatially resolved transcriptomics. *Nature Methods*, *18*(1), 9–14.

Matthews, B. W. (1975). Comparison of the predicted and observed secondary structure of T4 phage lysozyme. *Biochimica et Biophysica Acta (BBA)-Protein Structure*, *405*(2), 442–451.

Miller, B. F., Bambah-Mukku, D., Dulac, C., Zhuang, X., & Fan, J. (2021). Characterizing spatial gene expression heterogeneity in spatially resolved single-cell transcriptomic data with nonuniform cellular densities. *Genome Research*, *31*(10), 1843–1855.

Neal, R. M. (2003). Slice sampling. *The Annals of Statistics*, *31*(3), 705–767.

Neal, R. M., et al. (2011). MCMC using Hamiltonian dynamics. *Handbook of markov chain monte carlo*, *2*(11), 2.

Oh, T., Baek, S. H., Hwang, E.-H., An, Y. J., Kim, Y., Kim, D.-Y., & Hong, J. J. (2025). Spatiotemporal cellular dynamics of germinal center reaction in coronavirus disease 2019 lung-draining lymph node based on imaging-based spatial transcriptomics. *Laboratory Investigation*, *105*(1), 102180.

Perkins, N. J., & Schisterman, E. F. (2006). The inconsistency of "optimal" cutpoints obtained using two criteria based on the receiver operating characteristic curve. *American Journal of Epidemiology*, *163*(7), 670–675.

Qi, J., Zhou, Y., Zhao, Z., & Jin, S. (2021). Sdimpute: a statistical block imputation method based on cell-level and gene-level information for dropouts in single-cell rna-seq data. *PLoS Computational Biology*, *17*(6), e1009118.

Qin, H., Xiao, Q., Xie, Y., Li, D., Long, X., Li, T., Liu, Y., Chen, J., & Xu, F. (2023). The relationship between VEGF-460 polymorphism and cancer risk: A systematic review and meta-analysis based on 46 reports. *Medicine*, *102*(26), e34089.

Ruan, P., & Wang, S. (2021). DiSNEP: a disease-specific gene network enhancement to improve prioritizing candidate disease genes. *Briefings in Bioinformatics*, *22*(4), bbaa241.

Sant, P., Rippe, K., & Mallm, J.-P. (2023). Approaches for single-cell RNA sequencing across tissues and cell types. *Transcription*, *14*(3-5), 127–145.

Scimeca, M., Giannini, E., Antonacci, C., Pistolese, C. A., Spagnoli, L. G., & Bonanno, E. (2014). Microcalcifications in breast cancer: an active phenomenon mediated by epithelial cells with mesenchymal characteristics. *BMC Cancer*, *14*, 1–10.

Shang, L., & Zhou, X. (2025). Statistical identification of cell type-specific spatially variable genes in spatial transcriptomics. *Nature Communications*, *16*(1).

Sorzano, C. O. S., Vargas, J., & Montano, A. P. (2014). A survey of dimensionality reduction techniques. *arXiv preprint. arXiv:1403.2877*.

Ståhl, P. L., Salmén, F., Vickovic, S., Lundmark, A., Navarro, J. F., Magnusson, J., Giacomello, S., Asp, M., Westholm, J. O., Huss, M., et al. (2016). Visualization and analysis of gene expression in tissue sections by spatial transcriptomics. *Science*, *353*(6294), 78–82.

Sun, L., & Clayton, M. (2008). Bayesian analysis of crossclassified spatial data with autocorrelation. *Biometrics*, *64*(1), 74–84.

Svensson, V., Teichmann, S. A., & Stegle, O. (2018). Spatialde: identification of spatially variable genes. *Nature Methods*, *15*(5), 343–346.

Tang, W., Bertaux, F., Thomas, P., Stefanelli, C., Saint, M., Marguerat, S., & Shahrezaei, V. (2020). baynorm: Bayesian gene expression recovery, imputation and normalization for single-cell RNA-sequencing data. *Bioinformatics*, *36*(4), 1174–1181.

Vahid, M. R., Brown, E. L., Steen, C. B., Zhang, W., Jeon, H. S., Kang, M., Gentles, A. J., & Newman, A. M. (2023). High-resolution alignment of single-cell and spatial transcriptomes with CytoSPACE. *Nature Biotechnology*, *41*(11), 1543–1548.

Van Erp, S., Oberski, D. L., & Mulder, J. (2019). Shrinkage priors for Bayesian penalized regression. *Journal of Mathematical Psychology*, *89*, 31–50.

Wakefield, J., et al. (2013). *Bayesian and frequentist regression methods*, vol. 23. Springer.

Wang, J., Ma, A., Chang, Y., Gong, J., Jiang, Y., Qi, R., Wang, C., Fu, H., Ma, Q., & Xu, D. (2021). scgnn is a novel graph neural network framework for single-cell RNA-Seq analyses. *Nature Communications*, *12*(1), 1882.

Warburg, O. (1956). On the origin of cancer cells. *Science*, *123*(3191), 309–314.

Williams, C. G., Lee, H. J., Asatsuma, T., Vento-Tormo, R., & Haque, A. (2022). An introduction to spatial transcriptomics for biomedical research. *Genome Medicine*, *14*(1), 68.

Wong, K., Navarro, J. F., Bergenstråhle, L., Ståhl, P. L., & Lundeberg, J. (2018). ST Spot Detector: a web-based application for automatic spot and tissue detection for spatial transcriptomics image datasets. *Bioinformatics*, *34*(11), 1966–1968.

Zeng, H., Huang, J., Zhou, H., Meilandt, W. J., Dejanovic, B., Zhou, Y., Bohlen, C. J., Lee, S.-H., Ren, J., Liu, A., et al. (2023). Integrative in situ mapping of single-cell transcriptional states and tissue histopathology in a mouse model of Alzheimer's disease. *Nature Neuroscience*, *26*(3), 430–446.

Zhang, D., Schroeder, A., Yan, H., Yang, H., Hu, J., Lee, M. Y., Susztak, K., Xu, G. X., Feldman, M. D., et al. (2024). Inferring super-resolution tissue architecture by integrating spatial transcriptomics with histology. *Nature Biotechnology*, (pp. 1–6).

Zhang, Q., Jiang, S., Schroeder, A., Hu, J., Dai, D., Lee, E. B., Xiao, R., & Li, M. (2023). Leveraging spatial transcriptomics data to recover cell locations in single-cell RNA-seq with celery. *Nature Communications*, *14*(1), 4050.

Zhu, Q., Shah, S., Dries, R., Cai, L., & Yuan, G.-C. (2018). Identification of spatially associated subpopulations by combining scRNAseq and sequential fluorescence in situ hybridization data. *Nature Biotechnology*, *36*(12), 1183–1190.
